# Unravelling the Evolution of the Allatostatin-Type A, KISS and Galanin Peptide-Receptor Gene Families in Bilaterians: Insights from Anopheles Mosquitoes

**DOI:** 10.1371/journal.pone.0130347

**Published:** 2015-07-02

**Authors:** Rute C. Felix, Marlene Trindade, Isa R. P. Pires, Vera G. Fonseca, Rute S. Martins, Henrique Silveira, Deborah M. Power, João C. R. Cardoso

**Affiliations:** 1 Comparative Endocrinology and Integrative Biology, Centre of Marine Sciences, Universidade do Algarve, Campus de Gambelas, 8005–139, Faro, Portugal; 2 Centro de Malária e outras Doenças Tropicais, UEI Parasitologia Médica, Instituto de Higiene e Medicina Tropical, Universidade Nova de Lisboa, Rua da Junqueira 100, 1349–008, Lisboa, Portugal; University of Würzburg, GERMANY

## Abstract

Allatostatin type A receptors (AST-ARs) are a group of G-protein coupled receptors activated by members of the FGL-amide (AST-A) peptide family that inhibit food intake and development in arthropods. Despite their physiological importance the evolution of the AST-A system is poorly described and relatively few receptors have been isolated and functionally characterised in insects. The present study provides a comprehensive analysis of the origin and comparative evolution of the AST-A system. To determine how evolution and feeding modified the function of AST-AR the duplicate receptors in *Anopheles* mosquitoes, were characterised. Phylogeny and gene synteny suggested that invertebrate AST-A receptors and peptide genes shared a common evolutionary origin with KISS/GAL receptors and ligands. AST-ARs and KISSR emerged from a common gene ancestor after the divergence of GALRs in the bilaterian genome. In arthropods, the AST-A system evolved through lineage-specific events and the maintenance of two receptors in the flies and mosquitoes (Diptera) was the result of a gene duplication event. Speciation of *Anopheles *mosquitoes affected receptor gene organisation and characterisation of AST-AR duplicates (GPRALS1 and 2) revealed that in common with other insects, the mosquito receptors were activated by insect AST-A peptides and the iCa^2+^-signalling pathway was stimulated. *GPRALS1* and *2* were expressed mainly in mosquito midgut and ovaries and transcript abundance of both receptors was modified by feeding. A blood meal strongly up-regulated expression of both *GPRALS* in the midgut (*p* < 0.05) compared to glucose fed females. Based on the results we hypothesise that the AST-A system in insects shared a common origin with the vertebrate KISS system and may also share a common function as an integrator of metabolism and reproduction. Highlights: AST-A and KISS/GAL receptors and ligands shared common ancestry prior to the protostome-deuterostome divergence. Phylogeny and gene synteny revealed that *AST-AR* and *KISSR* emerged after *GALR* gene divergence. *AST-AR* genes were present in the hemichordates but were lost from the chordates. In protostomes, *AST-ARs* persisted and evolved through lineage-specific events and duplicated in the arthropod radiation. Diptera acquired and maintained functionally divergent duplicate *AST-AR* genes.

## Introduction

Type A allatostatins (AST-As) are a family of insect peptides with a conserved C-terminal FGL-amide motif. They were initially isolated from the cockroach *Diploptera punctata* [1,2] but are widespread in insects and are mainly detected in the brain and midgut [3–12]. AST-A peptides arise by proteolytic cleavage of a common prohormone precursor and a variable number of peptides of differing lengths have been identified [13–16]. In cockroaches (*D*. *punctata* [1,2], *Blattella germanica* [17], *Periplaneta americana* [2]), cricket (*Gryllus bimaculatus*) [18], locust (*Locust migratoria*) [19] and the termite (*Reticulitermes flavipes*) [20], AST-A peptides inhibit juvenile hormone (JH) secretion by the *corpora allata* (CA) but they have numerous other physiological roles including the regulation of food intake in many different insects [13,14,16,21–29]. In *B*. *germanica* injections of AST-A reduce food intake [21]. In *Drosophila melanogaster* genetic manipulation of neurons expressing AST-A repress food intake and responsiveness to sugar [23] and ablation of AST-A and its receptor (DAR-1) significantly reduce larval foraging behaviour in the presence of food [27]. The actions of AST-As on feeding are associated with their anti-myotropic actions on insect gut motility and regulation of digestive enzyme activity [29–35].

AST-A peptides activate specific G-protein coupled receptors (GPCRs), the insect allatostatin-A receptors (AST-ARs) that are considered orthologues of galanin receptors (GALR) in vertebrates [36–41]. In vertebrates, GALRs have a close evolutionary relationship with kisspeptin receptors (KISSR) and are activated by galanin (GAL) and spexin (SPX), peptides that are unrelated to insect AST-As [40–42]. AST-A peptide function is relatively well studied but the receptors have only been isolated in a few insect species and their evolution and function is unresolved [11,38,43–46]. In the fruit fly *D*. *melanogaster* two receptors, DAR-1 and DAR-2 have been de-orphanized [36,38,44,47,48] but in most insects only a single receptor gene exists [49,50]. The beetle *Tribolium castaneum* is the exception as it lacks both AST-A and the receptors [51–53]. In contrast, in the nematode, *Caenorhabditis elegans*, an orthologue of the insect AST-ARs was characterised (*npr-9*) [39], and two putative AST-A peptide encoding genes (*nlp-5* and *nlp-6*) were also identified [41,54,55]. All the studies of AST-A to date suggest that its role in feeding behaviour emerged early during its evolution and have probably been maintained during the Ecdysozoa radiation [14,22,25].

Functional specialisation of the AST-A system appears to have occurred in the insects. For example, in larval *D*. *melanogaster* DAR-1 is mainly present in the central nervous system (CNS) and DAR-2 is detected in the gut [56]. Comparison of AST-A activation of DAR-1 and DAR-2 reveals differences in binding and intracellular signalling in the presence of Pertussis toxin (PTX), an inhibitor of G_i_-type G-protein activity [47]. In the mosquito *Anopheles gambiae* (PEST strain) genome, duplicate AST-ARs also exist [50] and microarray data for blood fed females suggests that they are also functionally distinct as only the *D*. *melanogaster* DAR-1 orthologue is up-regulated 3 h after a blood meal [57].

The present study characterises the origin and evolution of AST-AR and their peptide ligands in arthropods and by isolating and characterizing the duplicate receptors in *Anopheles* mosquitoes determines if evolution modified receptor function. *Anopheles* mosquitoes are vectors of the malaria parasite and more than 400 species have been identified [58]. The genomes of *Anopheles* species are rapidly evolving and they have been used as models of how the environment and geographic isolation favour speciation and have modified gene structure and function [59–63]. Phylogeny coupled to gene synteny analysis revealed that the arthropod AST-ARs and AST-A peptides shared a common evolutionary origin with the KISS/GAL systems and that AST-AR and KISSR members probably emerged from the same gene after duplication of the AST-AR/KISSR/GALR ancestor. In arthropods, AST-ARs evolved under lineage-specific pressure and in the *Anopheles* mosquito speciation affected receptor gene evolution. Characterisation of the duplicate AST-ARs from *Anopheles coluzzii*, formerly known as *A*. *gambia*e M-form [59], revealed their sequences diverge and their response to a blood meal differs. The results of the present study are used to develop a model for the evolution of the AST-A system.

## Material and Methods

### *In silico* database mining

*AST-AR* genes were identified and retrieved from 21 arthropod genomes available in the Ensembl metazoan database (http://metazoa.ensembl.org/index.html, January 2014) by conducting similarity searches with the deduced mature sequence of *Drosophila melanogaster* DAR-1 (FBgn0028961) and DAR-2 (FBgn0039595) and using database gene annotations. The arthropod genomes analysed encompassed three different classes: Insecta, Arachnida and Branchiopoda. The representatives of the Insecta class included members of six orders: Diptera (*D*. *melanogaster*, *Megaselia scalaris*, *Anopheles gambiae*, *Anopheles darlingi*, *Aedes aegypti* and *Culex quinquefasciatus*); Lepidoptera (*Bombyx mori*, *Heliconius melpomene* and *Danaus plexippus*); Hymenoptera (*Nasonia vitripennis*, *Apis mellifera*, *Atta cephalotes* and *Solenopsis invicta*); Hemiptera (*Acyrthosiphon pisum* and *Rhodnius prolixus*); Phthiraptera (*Pediculus humanus*), and Coleoptera (*Tribolium castaneum* and *Dendroctonus ponderosae*). Other arthropod representatives included in this study were members of two Arachnidan orders: Ixodida (*Ixodes scapularis*) and Trombidiformes (*Tetranychus urticae)* and one representative of the Branchiopoda, *Daphnia pulex*. Searches for the arthropod *AST-A* gene were also performed using the deduced mature protein sequence of the *D*. *melanogaster* AST-A precursor (FBgn0015591) and genome annotations.

The reference mosquito genome used for *in silico* studies was the *A*. *gambiae* PEST strain, which is an M and S chimera [62,64,65]. The assemblies of 20 other *Anopheles* genomes were analysed and included several members of the *A*. *gambiae* complex (*A*. *arabiensis*, *A*. *gambiae* S-form, *A*. *merus*, *A*. *quadriannulatus*, *A*. *melas* and *A*. *coluzzii*, formerly known as *A*. *gambia*e M-form [59]) available from VectorBase (https://www.vectorbase.org/, March 2015).

### Sequence comparisons and phylogenetic analysis

Multiple amino acid sequence alignments of receptors were generated using ClustalW (v2) (http://www.genome.jp/tools/clustalw/) software. Conserved sequence motifs were identified and the percentage of receptor amino acid sequence similarities was calculated in GeneDoc [66].

Phylogenetic analysis of arthropod AST-ARs was performed using homologues retrieved from other invertebrates, the nematode *Caenorhabditis elegans* [39]; the lophotrochozoans, polychaete annelid worm (*Capitella teleta*), the owl limpet (*Lottia gigantean*) and the early deuterostomes, acorn worm (*Saccoglossus kowalevskii*), purple sea urchin (*Strongylocentratus purpuratus*), amphioxus (*Branchiostoma floridae*) and the tunicate (*Ciona intestinalis*). Deuterostome KISSR and GALR receptor sequences were obtained from [41] and [67]. Trees were constructed using an alignment of the deduced amino acid sequence from transmembrane (TM) region 1 to 7 (TM1 to 7) including intra and extracellular loops (S1 Table) submitted to ProtTest 2.4 to select the best statistical model to study receptor protein evolution according to the Akaike Information Criterion (AIC) [68].

Phylogenetic trees were constructed using maximum likelihood (ML) and neighbour-joining (NJ) methods and bootstrapped to assign measures of accuracy to the clades [69]. Trees were constructed with a total of 128 sequences and rooted with the vertebrate GPR151 receptor cluster (12 sequences). The ML analysis was built in PhyML 3.0 available from ATGC (http://www.atgc-montpellier.fr/phyml/) [70] using a JTT substitution model with the following parameters: 4 gamma distributed rate categories, a fixed proportion of invariant sites (0.009) and a fixed gamma shape parameter (1.294). Reliability for internal branching was assessed using 100 bootstrap replicates and the aLRT SH-like test [71]. Sequence data was also analysed using the NJ method [72] with 1000 bootstrap replicates in MEGA version 5.1 [73]. The NJ tree was constructed using the pairwise deletion for gaps/missing data treatment option and fixed gamma 4 distributed rate categories (gamma = 1.294) to account for heterogeneity across sites. The consensus trees obtained with ML and NJ analysis shared similar topologies.

### Gene synteny analysis

Gene synteny was carried out using the ENSEMBL BioMart comparative tool (http://metazoa.ensembl.org/biomart/martview/). The regions upstream and downstream of *A*. *gambiae AST-ARs* and *AST-A* locus were used to identify homologue genome regions in other insects (*D*. *melanogaster* and *T*. *castaneum*) and in the nematode *C*. *elegans* genomes. Genes in a 10 Mb segment flanking *GPRALS1* and *GPRALS2* in *A*. *gambiae* chr 2R and a 3 Mb region flanking *AST-A* in *A*. *gambiae* chr 2R were used to search *D*. *melanogaster*, *T*. *castaneum* and *C*. *elegans* genomes. Conserved genes flanking *AST-AR* and *AST-A* across invertebrates were used to establish synteny with human *KISSR* and *GALR* genome regions. The identity and evolutionary relatedness of the flanking genes identified was confirmed using sequence similarity searches against the human, insect and nematode genome assemblies and confirmed with phylogenetic analysis when necessary. Orthologues of the genes identified in the human *KISSR* and *KISS/GAL/SPX* paralogon were also mapped in the insect and nematode genomes [42,74].

### Ethics statement

All animal experiments were performed at the Centro de Malária e outras Doenças Tropicais, Instituto de Higiene e Medicina Tropical (IHMT, Lisbon). This study was approved by the IHMT committee on the ethics for animal experiments and by the Direção-Geral de Veterinária, Ministério da Agricultura do Desenvolvimento Rural e das Pescas, Portugal licences (id approvals: 023351 and 023355). Animal experiments were carried out in strict accordance with Portuguese law and following the guidelines for care and use of laboratory animals. All the authors that performed animal manipulations were licensed to conduct research using laboratory animals.

### Mosquito rearing

Mosquitoes from a laboratory colony of *A*. *gambiae* (Yaoundé strain), recently renamed *A*. *coluzzii* [59] were maintained under standard insectary conditions. Temperature was maintained at 26 ± 1 °C, humidity at 75% and a 12:12 h light:dark cycle were used in all experiments. Adult mosquitoes were allowed to feed *ad libitum* on a 10% glucose solution.

### Feeding and tissue collection

*A*. *coluzzii* females were food deprived for 3 h and then fed for 30 min on either a 10% glucose solution or a blood meal (obtained from anaesthetized 6–8 week old CD1 mice, *Mus musculus*). Whole mosquito females and dissected tissues (midguts, fat bodies, ovaries and heads) of 20 glucose and/or blood fed mosquitoes from three independent experiments were collected and stored in RNA later at -20°C for RNA extraction.

### RNA extractions and cDNA synthesis

Total RNA (tRNA) from whole *A*. *coluzzii* or specific tissues was isolated using a kit (total RNA Kit I, Omega, VWR, Portugal) and genomic DNA was eliminated by treating with 1 U DNase (DNA-free Kit, Ambion, UK) for 30 min at 37 °C. DNase treated tRNA (500 ng) was denatured at 65 °C for 5 min, quenched on ice for 5 min and used for cDNA synthesis in a 20 μl reaction volume containing 10 ng of pd(N)6 random hexamers (GE Healthcare, UK), 2 mM dNTPs, 100 U of MMLV-RT and 20 U RNasin Plus RNase inhibitor (Promega, Spain). The cDNA was synthesized for 10 min at 25 °C followed by 60 min at 42 °C and 70 °C for 10 min. Due to the small amounts of RNA extracted from the ovaries, cDNA synthesis was performed using a single pool of RNA extracted from the 3 experimental groups. The quality of the synthesised cDNA was assessed by PCR amplification of a reference gene, ribosomal protein S7 sub-unit. The thermocycle used was: 95°C for 3 min; 35 cycles of 95°C for 30 s, 60°C for 30 s, 72°C for 30 s, followed by 72°C for 5 min. PCR reactions were carried out in a final reaction volume of 10 μl and contained 1.5 mM MgCl_2_ (Thermo Scientific, Portugal), 0.2 mM dNTP’s (GE Healthcare, Spain), 0.25 μM of gene specific primer pairs (S2 Table) and 0.5 U of DreamTaq DNA Polymerase (5 U/μl, Thermo Scientific, Portugal).

### Quantitative Polymerase Chain Reaction (q-PCR)

Quantitative real-time PCR (q-PCR) was used to quantify the expression of AST-ARs and of AST-A in female *A*. *coluzzii* midgut, fat body, head and ovaries when feed with glucose and blood meals. Specific primers were designed using the cloned receptor transcripts (S2 Table). Primers to amplify *AST-A* were designed based on the sequence retrieved from *A*. *gambiae* PEST AGAP003712 that is 100% identical to the EST clone (CR530883) isolated from a head cDNA library of a species from the *A*. *gambiae* complex. Expression of zinc carboxypeptidase A1 (*CP*, AGAP009593, a gut specific marker of the digestive process) [75] and the vitellogenin-1 precursor (*Vtg*, AGAP004203, a protein biomarker of egg production [76]) were also analysed.

Duplicate q-PCR reactions (< 5% variation between replicates) were amplified in a StepOne Plus Real-Time PCR Detection system (Applied Biosystems, Portugal) for 96-well microplates (Bio-Rad, Portugal). Analysis was performed in a final reaction volume of 10 μl that contained 300 nM of forward and reverse primer, SsoFast EvaGreen supermix (Bio-Rad, Portugal) and 2 μl of template cDNA (1:5). Optimized cycling conditions consisted of 95°C for 30 s, followed by 45 cycles of 95°C for 5 s and 10 s at the appropriate annealing temperature for primers. PCR reactions included a standard curve prepared from the purified PCR product of each target template. Melting curves were performed to detect primer dimers and negative control reactions were included to rule out genomic contamination. PCR reaction efficiencies and r^2^ (coefficient of determination) were established for each target gene (S2 Table). Target transcript normalisation was performed using the geometric mean of two reference genes: ribosomal S7 subunit (*S7*, AGAP010592) [77,78] and mitochondrial solute carrier family 25 (*MC*, AGAP001297) [79].

### Receptor cloning and mammalian cell transfections

The full-length of *A*. *coluzzii GPRALS1* and *GPRALS2* were amplified by PCR using proofreading DNA polymerase (iProof, BioRad, Portugal) and the specific primers designed using the sequence predicted in ENSEMBL (AGAP003658, *GPRALS1* and AGAP001773, *GPRALS2*; S2 Table). Receptors were amplified from cDNA obtained from whole female *A*. *coluzzii* using *Pfu* proofreading DNA polymerase (Promega, Spain). The thermocycle used was: 2 min at 95 °C, 35 cycles (95 °C for 1 min, the appropriate annealing temperature (°C) for 45 s, 72 °C for 4 min) and a final extension step of 10 min at 72 °C. PCR products were sequenced to confirm their identity and cloned into pGEM T easy vector (Promega). The purified PCR products were ligated into pcDNA3.1/V5-His TOPO TA expression vector (Invitrogen, USA). The complete *D*. *melanogaster* DAR-2-RA (FBtr0085316) was also isolated and amplified from adult cDNAs and cloned into a HindIII/NotI digested pcDNA3.1/V5-His TOPO TA vector.

The amplified insect receptors included the initiation and termination codons but were not cloned in frame with vector tag proteins. The recombinant constructs were used to transfect mammalian CHO cells that had been maintained in complete Dulbecco’s modified Eagle’s medium (DMEM, Sigma, Spain) containing 4.5 g/L glucose, 110 mg/L sodium pyruvate and L-glutamine and supplemented with 10% sterile foetal bovine serum and 0.1% penicillin: streptomycin antibiotic mix (10.000 U: 10 mg/ml, Sigma) and 250μg/ml sterile filtered amphotericin B solution (1:100 dilution, Sigma, Spain). One day prior to transfection, 2–3 x10^5^ cells were seeded into 6 well plates (Sarstedt, Portugal) and transfected using Fugene 6 reagent (1: 6 DNA: Fugene, Roche, Germany) according to the manufacturer’s protocol. The efficiency and success of cell transfection was estimated by performing a simultaneous transfection with a plasmid encoding a fluorescent protein. 72 h after incubation, transformed cells were selected by supplementing medium with 800μg/ml of the antibiotic Geneticin (G418 sulphate, GibcoBRL, USA) and cell recovery was monitored daily and the medium changed until no cell death was observed. Establishment of AST-AR CHO stable cell lines were confirmed by PCR using receptor specific primers.

### Peptides

The *A*. *gambiae* AST-A1 (SPKYNFGL-NH_2_) and AST-A2 (LPHYNFGL-NH_2_) peptides were chemically synthesized. Peptide sequences were deduced from AGAP003712 by comparing with *D*. *melanogaster* (FBgn0015591) and the *A*. *aegypti* (U66841) AST-A precursors [80,81]. *A*. *gambiae* peptides were 100% identical to the deduced peptide sequence of *A*. *coluzzii* (Yaoundé strain) transcripts and to the gene homologues of other members of the *A*. *gambiae* complex and were designated Ano_AST-A1 and Ano_AST-A2. Peptides with a purity > 95% (ChinaPeptides, China) were diluted in 1 × PBS buffer for the cAMP and iCa^2+^ assays. Other peptides used included: the German cockroach *Blattella germanica* BLAST-2 peptide (DRLYSFGL-NH_2_, kindly donated by Dr. Maria Dolors Piulach, CSIC-UPF, Barcelona, Spain [17]), 1–29 rat galanin (Sigma-Aldrich, Spain) and the vertebrate KISS peptides, sea bass KISS 1–10 (YNLNSFGLRY-NH_2_) and KISS 2–10 (FNFNPFGLRF-NH_2_) (kindly donated by Dr Ana Gomez, CSIC-IATS, Spain [82]).

### Receptor signalling

GPRALS1 and GPRALS2 stable CHO cell lines were stimulated with Ano_AST-A1 and Ano_AST-A2 peptides, the *B*. *germanica* BLAST-2 peptide and rat GAL and sea bass KISS peptides and iCa^2+^ release and cAMP production were measured. The *B*. *germanica* BLAST-2 peptide shared an identical amino acid (aa) sequence with *D*. *punctata* AST-5 that stimulates iCa^2+^ mobilization by both *D*. *melanogaster* receptors [47].

For iCa^2+^ release (Relative Fluorescent Units, RFU) a Ca^2+^ sensitive fluorescent dye Fluo-4 NW (Molecular Probes, Invitrogen) was used. Approximately 50,000 cells were assayed per well and the variation in fluorescence after addition of increasing peptide concentrations (1 nM to 1 μM, diluted in the assay buffer) was measured every 5s over a total of 2 min and fluorescence was excited using a 485/20 nm filter and captured with a 528/20 nm filter in a Biotek Synergy 4 plate reader (Biotek, USA). Background RFU of transfected cells was measured prior to peptide stimulation. The controls consisted of transfected cells exposed to carbachol (100 nM; Sigma, Spain) and non-transfected CHO cells stimulated with 1 μM of each peptide (negative control).

The amount of cAMP produced was determined using a competitive immunoassay with a cryptate labelled anti-cAMP antibody (Cisbio, France) and following the manufactures protocol. Approximately 15,000 cells were assayed per well and peptide incubations were performed in a final reaction volume of 20 μl in white 384 well small Volume HiBase Polystyrene microplates (Greiner, Germany). Prior to the assay, cells were ressuspended in 1 x PBS with 1 mM of 3-isobutyl-1- methylxantine (IBMX, Sigma) and incubated for 5 min at 37°C. Peptides were diluted in 1 x PBS/ 1 mM IBMX and were added to the cells for 30 min at 37°C in a CO_2_ incubator before measuring with 620/10 and 665/8 nm filters in a Biotek Synergy 4 plate reader (Biotek, USA). All assays were performed in triplicate on three independent occasions.

### Statistical analysis

Quantitative expression data is presented as mean ± SEM of cDNA from 3 independent experiments analysed in duplicate. Significant changes in transcript expression were assessed using a nonparametric Mann-Whitney two-tailed test. Receptor activation is presented as the mean ± SEM of three independent experiments carried out in triplicate and statistical significance was assessed using a Kruskal-Wallis test with Dunn’s Multiple Comparison Test. All the analyses were performed in Prism GraphPad version 5 and statistical significance was considered at *p* < 0.05.

## Results

### AST-AR and peptides in arthropods

**Fig 1:**
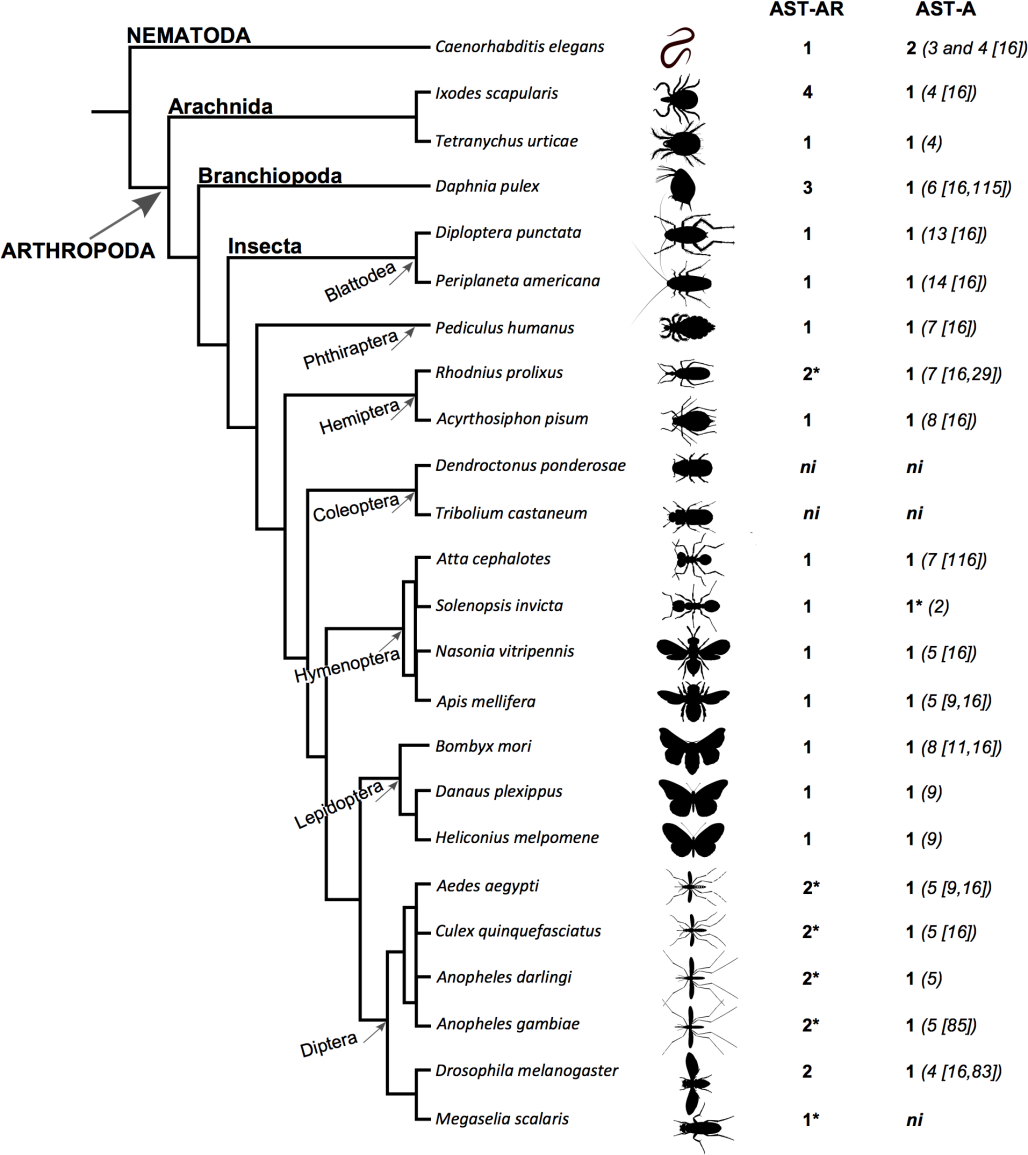
Number of predicted AST-AR and peptide genes identified in arthropods and *C*. *elegans*. Accession numbers are available in S1 Table. The number of AST-A peptides is indicated within brackets and references are provided. The *T*. *urticae*, *D*. *plexippus*, *H*. *melpomene*, *S*. *invicta* and *A*. *darlingi* AST-A peptides were predicted by comparison with the insect homologues and identification of the C-terminal FGL-amide motif. * indicates species in which a putative *AST-AR* pseudogene (orthologue of the third Culicidae *AST-AR* gene) was identified. Data from *D*. *pulex* and *A*. *cephalotes* obtained from [115, 116].

Searches performed in arthropod genomes identified 30 putative *AST-AR* and 18 putative *AST-A* genes (Fig 1, S1 Table). The results obtained indicated that receptor gene number across arthropods was very variable but that the number of genes encoding the peptides was conserved. In common with *Drosophila melanogaster*, two receptor genes were identified in Culicidae including the malaria vector *Anopheles gambiae* PEST strain, the yellow fever mosquito *Aedes aegypti* and the southern house mosquito *Culex quinquefasciatus*. In *Anopheles darling* genome two receptors homologues of the *A*. *gambiae* AST-AR genes were identified. In other Diptera representatives (11 *Drosophila* species: *D*. *ananassae*, *D*. *erecta*, *D*. *grimshawi*, *D*. *mojavensis*, *D*. *pseudoobscura*, *D*. *persimilis*, *D*. *sechellia*, *D*. *simulans*, *D*. *virillis*, *D*. *willistoni* and *D*. *yakuba*) two receptors were also identified (data not shown). The exception was the humpbacked fly *Megaselia scalaris* (Phoridae family) for which a single receptor that shared highest sequence similarity with *D*. *melanogaster* DAR-2 receptor was retrieved. It remains to be established if the failure to identify two AST-AR genes in *M*. *scalaris* was the consequence of the incomplete assembly of its genome. Two receptors were also identified in the genome of the kissing bug *Rhodnius prolixus* but in the remaining insect species a single receptor gene was identified: silkworm *Bombyx mori*, monarch butterfly *Danaus plexippus*, postman butterfly *Heliconius melpomene*, honey bee *Apis mellifera*, jewel wasp *Nasonia vitripennis*, red fire ant *Solenopsis invicta*, leaf-cutter ant *Atta cephalotes*, pea aphid *Acyrthosiphon pisum* and human lice *Pediculus humanus*. The exception was the Coleopterans; no *AST-AR* genes were retrieved from the genome of the red flour beetle *Tribolium castaneum* or the mountain pine beetle *Dendroctonus ponderosae*. In the branchiopod *Daphnia pulex*, 3 genes were recovered. In the arachnidan *Ixodes scapularis* 4 *AST-AR* genes were identified and in the red spider mite *Tetranychus urticae* only a single receptor gene was identified (Fig 1).

In the genomes of *A*. *gambiae* (AGAP001774) and *A*. *aegypti* (AAEL006077) a third *AST-AR* gene that mapped close to, and was more like *GPRALS2* but had a different orientation (antisense) was found. Orthologues were identified in *A*. *darlingi* (deduced from Scaffold_1464) and *C*. *quinquefasciatus* (CPIJ011118) and also in the genomes of *M*. *scalaris* (MESCA004796) and *R*. *prolixus* (RPRC004705) (S1 Table). The predicted insect receptor sequences encoded 3 or less TM domains and were excluded from the analysis. Despite strenuous efforts it was not possible to identify full-length genes and the sequences may represent pseudogenes arising from species-specific genome events (S1 Table).

**Fig 2:**
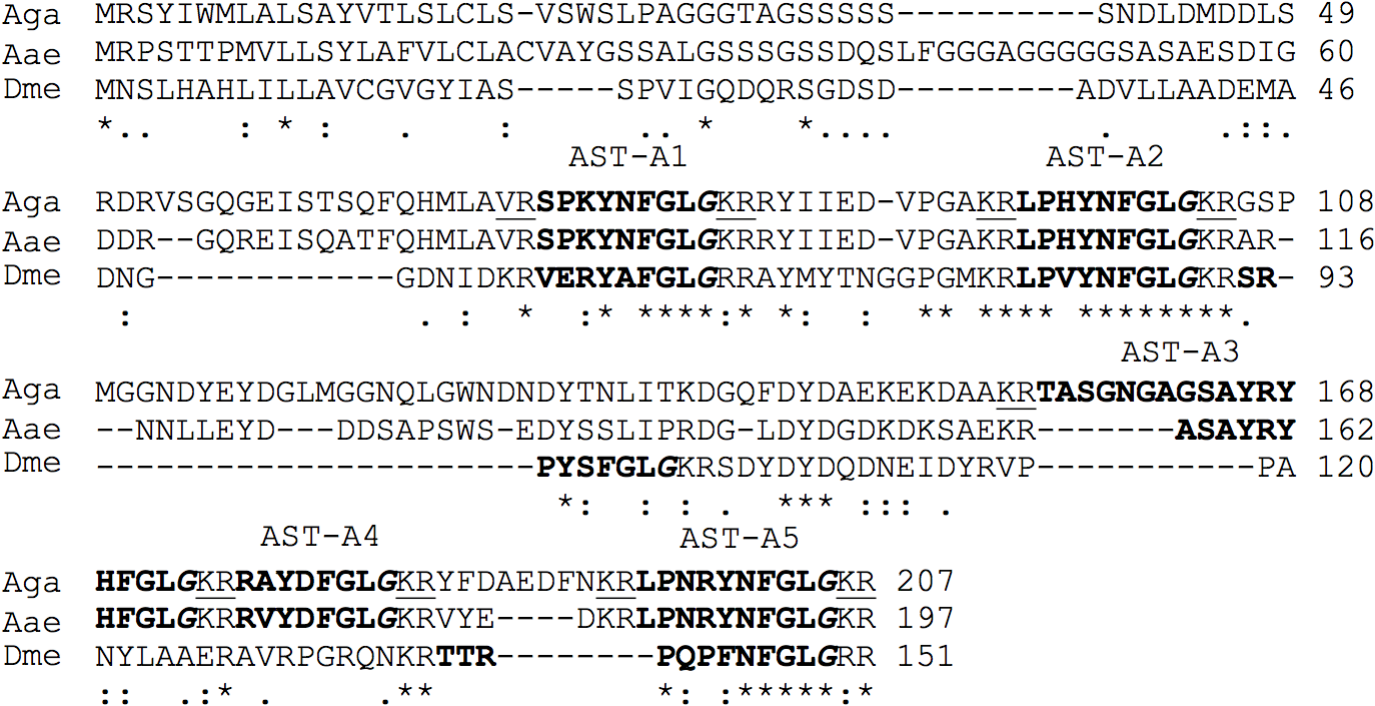
AST-A peptide precursor in *A*. *gambiae*. The deduced sequence of AST-A in *A*. *gambiae* (Aga, PEST) was obtained from the AGAP003712 gene and confirmed using EST data. The *A*. *aegypti* (Aae, AAEL015251,[81]) and *D*. *melanogaster* (Dme, FBgn0015591,[48]) orthologues were used for comparisons. The predicted mature peptides are highlighted in bold and the Gly residues processed to the C-terminal amide in mature AST-A’s are indicated in italics.

In arthropods a single *AST-A* gene was identified in the genomes of all species analysed with the exception of the two beetle genomes that lacked the genes (Fig 1). The number of mature AST-A peptides was highly variable across insects. Cockroaches had the most numerous AST-A (13 in *Diploptera punctata* and 14 in *Periplaneta americana* [16]) and the Diptera and Arachnida had the fewest AST-A (4 peptides *in D*. *melanogaster* [16,83] and *Ixodes scapularis* [16] and 5 peptides in mosquitoes [9,16]) (Fig 2).

### Phylogeny of AST-AR

**Fig 3:**
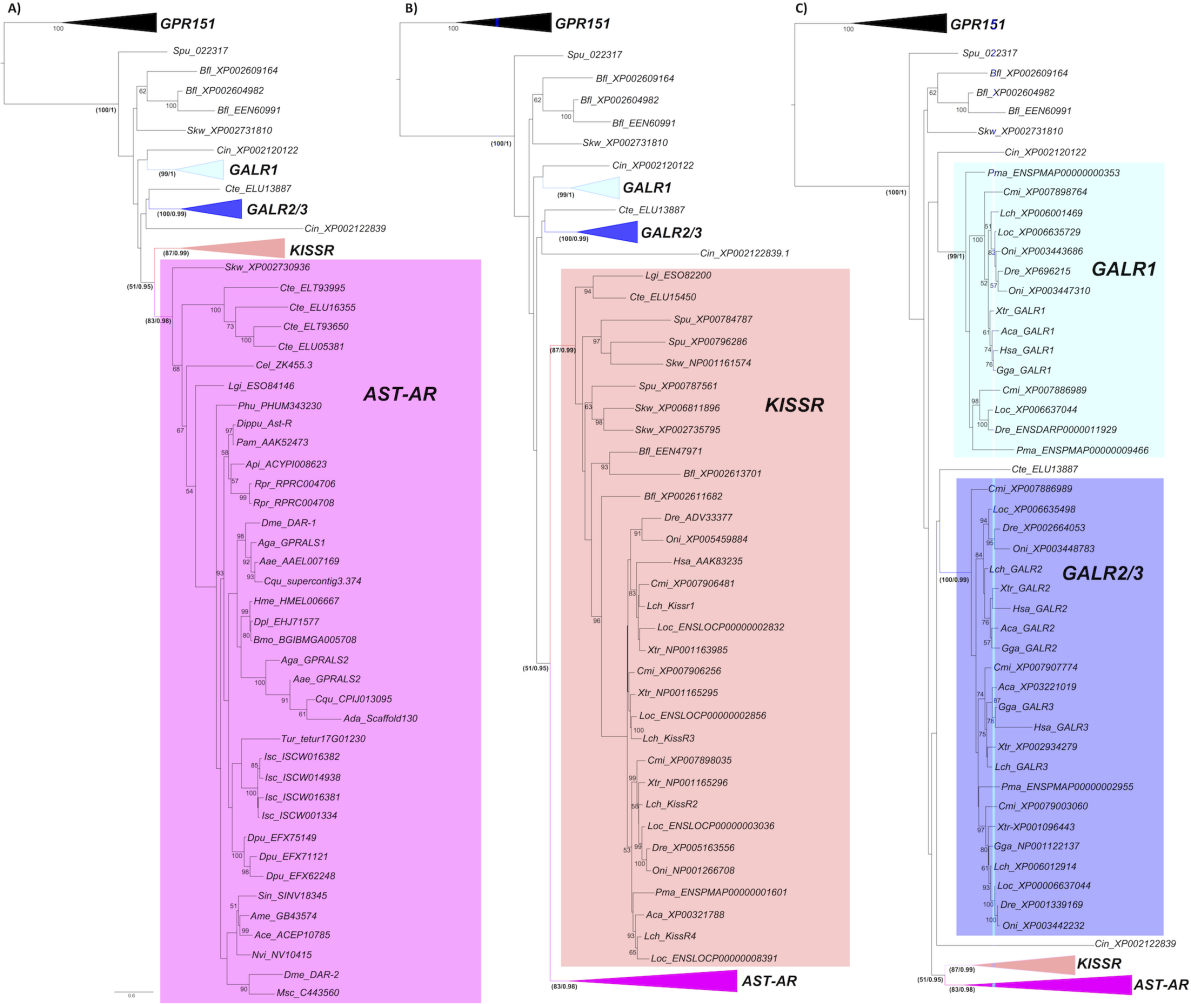
Phylogeny of the AST-AR with the KISSR and GALR. Phylogenetic analysis was performed using the ML method and three subsets of the same phylogenetic tree showing the expansion of the different family members (A, B and C) are represented to facilitate interpretation. Only bootstrap support values above 50% are indicated. In the most important receptor family nodes statistical support was established using the aLRT SH-like test and is indicated (bootstrap method/ aLRT SH-like test). The deduced *A*. *darlingi* (Scaffold_325) was not used, as the receptor sequence was very incomplete and only 3 TM domains were predicted. The phylogenetic tree was rooted with the vertebrate GPR151 cluster (12 sequences). Species names and accession numbers of the receptor genes are available in S1 Table.

Phylogenetic analysis suggested that in arthropods gene duplications and deletions affected AST-AR evolution. Orthologues of *D*. *melanogaster* DAR-1 in other Diptera were highly conserved but the duplicate receptors were highly divergent (Fig 3). A cluster of receptors that included DAR-1 and mosquito orthologues was identified but no equivalent cluster existed for DAR-2. In contrast, species-specific expansion of AST-ARs gene number occurred in *R*. *prolixus* (2 receptors), *D*. *pulex* (3 receptors) and *I*. *scapularis* (4 receptors) (Fig 3A). The tree topology of arthropod AST-ARs with homologues in other metazoans including the nematode *Caenorhabditis elegans*, the annelid, *Capitella teleta*, the mollusc, *Lottia gigantea* and the early deuterostome *Saccoglossus kowalevskii* suggested that they all shared a common ancestor.

The arthropod and other invertebrate AST-ARs tended to cluster in the phylogenetic trees with the protostome and deuterostome KISSR group rather than the GALRs (Fig 3). Paradoxically, the dipteran receptors (*D*. *melanogaster* and *A*. *gambiae*) shared slightly higher sequence identity/similarity with human GALR1 compared to KISSR1 (Table 1). The KISSR (Fig 3B) and the GALR (Fig 3C) clusters contained representatives from several vertebrate and invertebrate lineages including annelids, mollusc and early deuterostomes.

**Table 1:** Sequence identity and similarity of insect AST-ARs with human GALR1 and KISSR1.

	Dme_DAR-1	Bmo_BAR	Dippu_AstR	Pam_AstR	Aco_GPRALS2	Dme_DAR-2	Aae_AAEL007169	Cqu_CPIJ013095	Hsa_GALR1	Hsa_KISSR1
Aco_GPRALS1	59%	54%	48%	49%	48%	39%	66%	31%	29%	25%
	68%	64%	61%	60%	59%	54%	75%	37%	46%	41%
Dme_DAR-1		47%	45%	45%	43%	35%	54%	28%	27%	23%
		59%	59%	58%	57%	51%	63%	37%	44%	37%
Bmo_BAR			51%	51%	46%	38%	57%	32%	29%	26%
			64%	65%	59%	52%	68%	39%	46%	41%
Dippu_AstR				84%	42%	35%	47%	26%	26%	24%
				88%	54%	51%	60%	34%	45%	39%
Pam_AstR					43%	36%	48%	26%	26%	24%
					55%	51%	59%	34%	44%	39%
Aco_GPRALS2						44%	49%	39%	27%	23%
						61%	62%	46%	48%	40%
Dme_DAR-2							43%	27%	29%	25%
							59%	36%	50%	41%
Aae_AAEL007169								34%	31%	25%
								40%	49%	40%

Percentages were calculated using the full-length amino acid sequence of the receptors.

### AST-AR and peptide gene synteny

**Fig 4:**
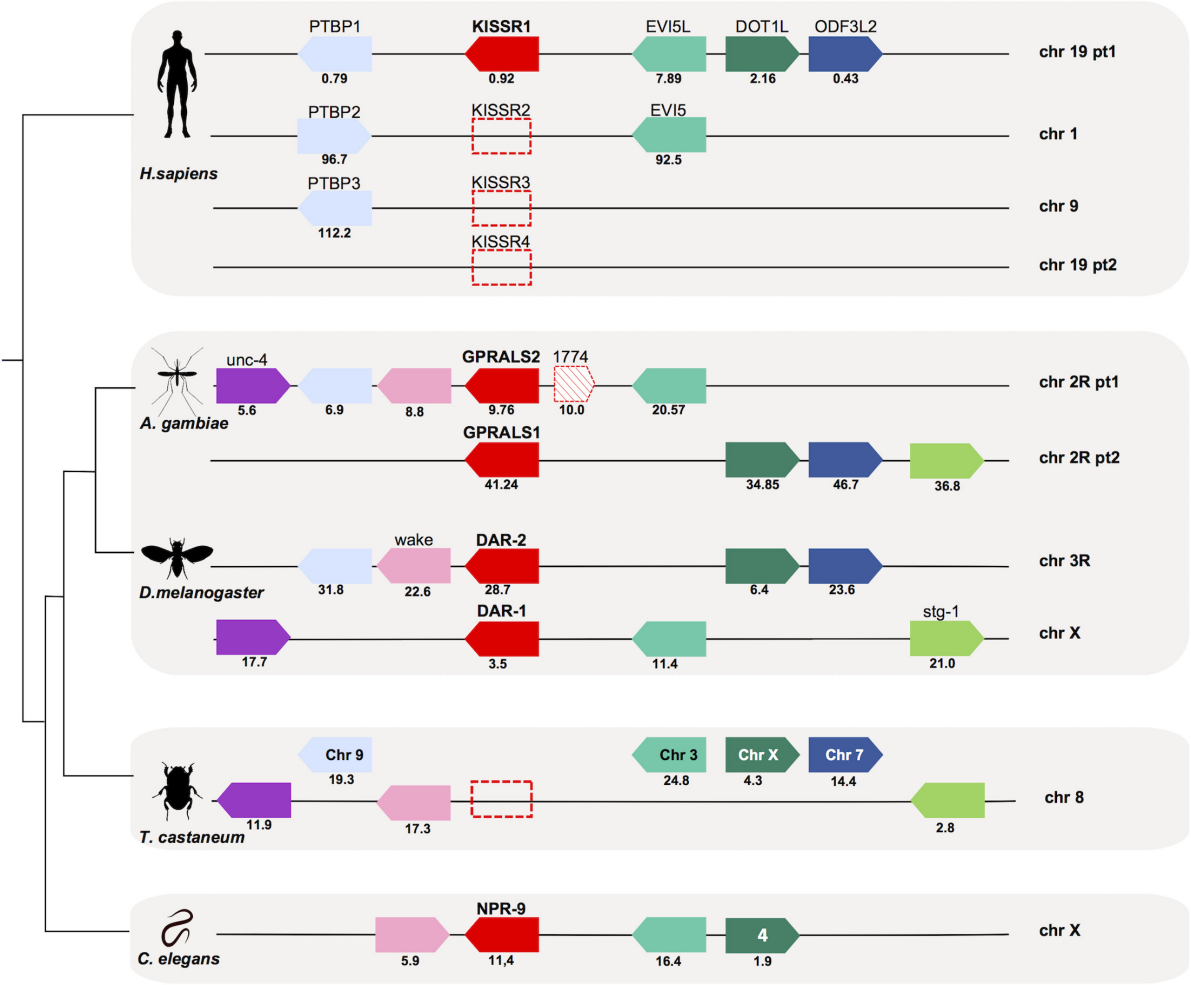
Conserved gene synteny of the *A*. *gambiae*, *D*. *melanogaster* and *C*. *elegans AST-AR* genome regions with the human *KISSR1* chromosomes. Conservation for *T*. *castaneum* is also shown. Horizontal lines represent chromosome fragments and block arrows indicate genes and orientation in the genome. Orthologue genes are represented in the same colour and their position (Mb) is indicated. An arrow with red stripes represents the putative *AST-AR* pseudogene (AGAP001774) localized near *GPRALS2*. Dotted boxes represent the absent human *KISSR* genes (that emerged during early vertebrate tetraploidizations) [67,74] and the *T*. *castaneum AST-AR* gene. Note that the mosquito 2R and human ch19 have been divided into two parts (pt1 and pt2) to facilitate visualization. Only shared genes are represented. The number of family members that map to the same chromosome is indicated and the closest to *AST-AR* and *KISSR1* is represented. A full description of gene families and names and accession numbers is given in S3 Table.

**Fig 5:**
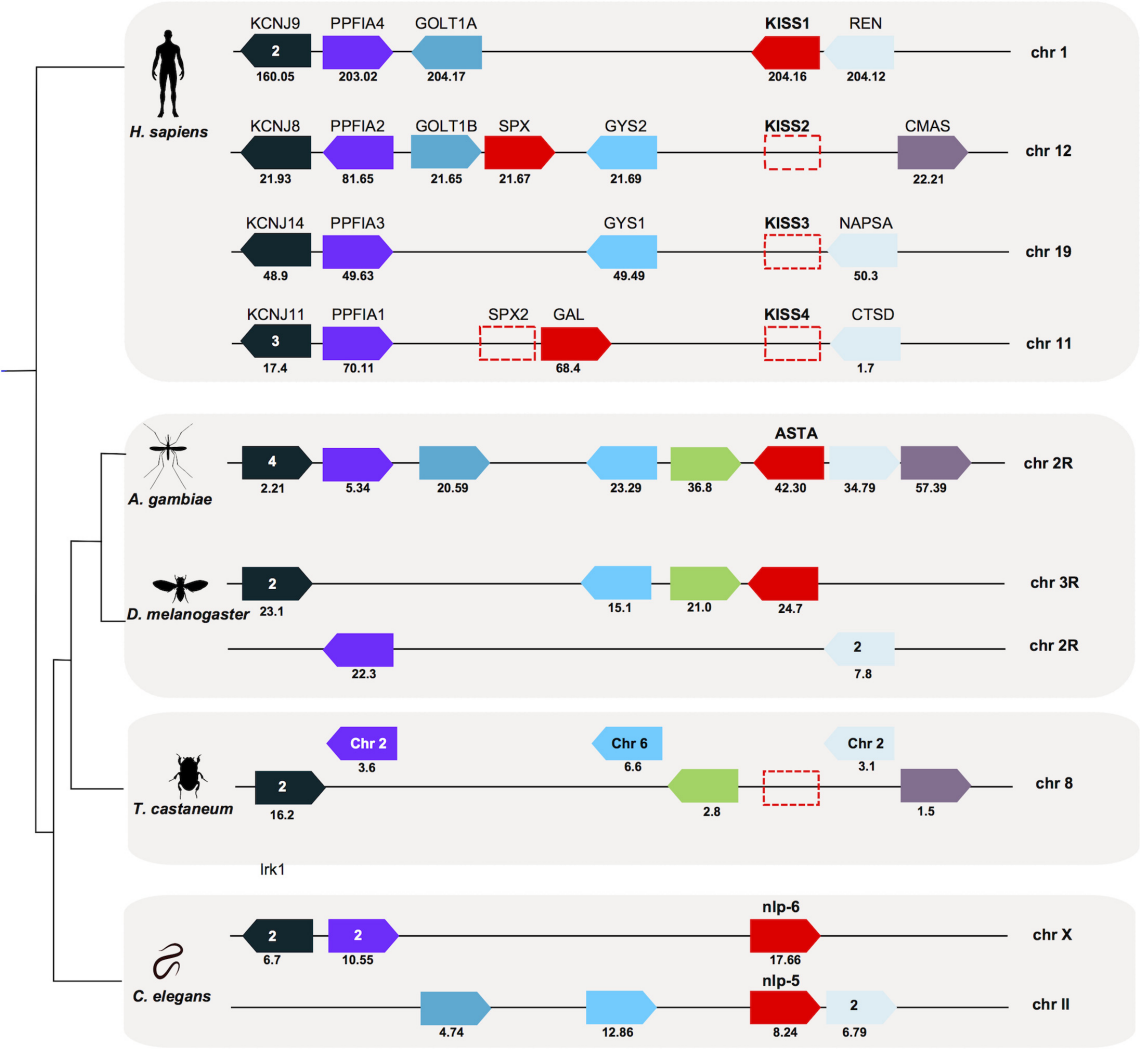
Conserved gene synteny of the genome regions containing *AST-A* gene in *A*. *gambiae*, *D*. *melanogaster* and *C*. *elegans* (*npl-5* and *npl-6*) compared to the human *KISS/GAL/SPX* chromosomes. The gene homologues in *T*. *castaneum* are also represented. Horizontal lines indicate chromosome fragments and coloured arrow identify genes and their orientation in the genome. Orthologue genes are indicated in the same colour and their positions are indicated below (Mb). Dotted boxes represent the absent human *KISS* and *SPX2* genes (that emerged during early vertebrate tetraploidizations) [42,74] and the *T*. *castaneum AST-A* gene. Only shared gene family members are represented. The number of family members that map to the same chromosome is indicated and those closest to *AST-A* and *KISS/GAL/SPX* are represented. A full description of gene families and names and accession numbers is given in S4 Table.

The gene environment of insect receptor and peptide genes was compared with *C*. *elegans* and human (Figs 4 and 5). The genes in linkage with *AST-AR* in *A*. *gambiae* and *D*. *melanogaster* were compared to the homologue genomic regions of human *GALR (GALR1*, chr 18; *GALR2*, chr 17 and *GALR3*, chr 22), human *KISSR1* (chr 19) and *C*. *elegans npr-9* (chr X) (Fig 4, S3 Table). In *A*. *gambiae GPRALS1* and *GPRALS2* genes were localised on chr 2R, while in the *D*. *melanogaster* they mapped to chr X (*DAR-1*) and chr 3R (*DAR-2*), although gene synteny was retained. The genome arrangement of *A*. *gambiae* and *D*. *melanogaster* chromosome regions containing *AST-ARs* suggested that they underwent distinct evolutionary pressure after gene duplication. The conserved gene linkage between the insect and the nematode *C*. *elegans* orthologue regions suggested that duplication of *AST-AR* occurred after the divergence and radiation of the nematodes.

None of the genes flanking insect *AST-ARs* were identified in the human *GALRs loci*. In contrast, neighbouring genes that flanked protostome *AST-AR* genes mapped to the human *KISSR1* chromosome paralogon (Fig 4). Members of 4 gene families (Polypyrimidine tract binding protein, *PTBP*; ecotropic viral integration site 5 proteins, *EVI5*; DOT1-like histone H3K79 methyltransferase proteins, *DOT1L*; and outer dense fiber of sperm tails 3 protein, *ODF3L*) flanked the human *KISSR1* gene on chromosome 19, *A*. *gambiae AST-ARs* on chr 2R, and *D*. *melanogaster DAR-1* on chr X and *DAR-2* on chr 3R. The *AST-AR* genome region in the nematode *C*. *elegans* contained members of 3 gene families linked to human *KISSR1* and insect *AST-AR* (Fig 4). The conserved gene environment that flanked *AST-ARs* in *C*. *elegans*, *A*. *gambiae*, *D*. *melanogaster* and *KISSR1* in human was absent from the *T*. *castaneum* genome (Fig 4). The genes in linkage with *AST-AR* in Diptera were distributed between chromosome 8, 9, 7, 3 and X in *T*. *castaneum* that lacks the *AST-AR* genes. The conservation on the beetle chr 8 of a greater number of genes from the dipteran *AST-AR* bearing chromosomes suggests it may be the homologue chromosome.

The genome region of *AST-A* genes in *A*. *gambiae* and *D*. *melanogaster* was also compared with the human *KISS/GAL/SPX* paralogon (chr 1, 11, 12 and 19) [42,74] and with the *C*. *elegans* chromosome that contained the *npl-5* (chr II) and *npl-6* (chr X) genes (Fig 5, S4 Table). In *A*. *gambiae*, *AST-A* shared the same chromosome localisation as the *GPRALSs* (chr 2R) and mapped closest to *GPRALS1*. A similar situation occurred in *D*. *melanogaster* and *C*. *elegans* and the gene encoding the peptide was also localised on the same chromosome as the receptors. In *D*. *melanogaster*, *AST-A* mapped near *DAR-2* on chr 3R and in *C*. *elegans* near *npr-9* on chr X. Members of metazoan gene families (inward rectifying potassium channel superfamily, *KCNJ*; LAR protein-tyrosine phosphatase-interacting protein, *PPFIA*, *PTPRF* and *Liprin*; Golgi Transport *GOLT*; glycogen synthase, *GYS*; aspartic protease family, *REN*, *NAPSA*, *CTSD*, *CathD*; and members of the N-acylneuraminate cytidylyltransferase, *CMAS*) were conserved in the region flanking the *AST-A* gene in *A*. *gambiae*, *D*. *melanogaster* and the human *KISS/GAL/SPX* paralogon. Representatives of five genes families that were in linkage with human *KISS/GAL/SPX* and insect *AST-A* were split between chr X that contained *npl-6* and chr II that contained *npl-5* in *C*. *elegans*. Conservation of genes that flanked *AST-A* and *KISS/GAL/SPX* genes suggests that they shared a common evolutionary origin (Fig 5). In *T*. *castaneum* these genes mapped to different chromosomes including chr 8.

### *AST-ARs* and *AST-A* genes in *Anopheles* mosquitoes

*GPRALS1* (1137 bp) and *GPRALS2* (1080 bp) were isolated from *A*. *coluzzii* whole female cDNA and the deduced proteins shared 48% aa identity. *A*. *coluzzii* GPRALS1 shared greatest aa sequence identity with *D*. *melanogaster* DAR-1 (59%) and with the orthologues from other arthropods such as *Bombyx mori* (54%), *Diploptera punctata* (48%) and *Periplaneta americana* (49%). The deduced protein sequence of *A*. *gambiae* GPRALS2 shared 43% and 44% aa identity with *D*. *melanogaster* DAR-1 and DAR-2, respectively (Table 1).

In the *A*. *gambiae* genome assembly, the two *AST-AR* genes had a different gene organisation and number of predicted transcripts. Two alternative transcripts of the same length (*GPRALS1-RA* and *GPRALS1-RB*) were predicted that shared 7 common exons but had a different exon 1 (S5 Table). The predicted *GPRALS2* gene structure was more complex and composed of 10 exons and alternative splicing was predicted to generate three almost identical transcripts: *GPRALS2-RA*; *GPRALS2-RB* and *GPRALS2-RC*. The transcripts shared the first exon but alternative splicing of 3 consecutive tandem duplicated clusters of three exons generated three predicted proteins that shared 96–99% aa identity. To confirm gene predictions ESTs for *A*. *gambiae* were analysed and a partial clone (BX620556) was identified that was identical to *GPRALS2-RC*. Other ESTs identified were very incomplete and the existence of multiple transcripts remains to be confirmed.

**Fig 6:**
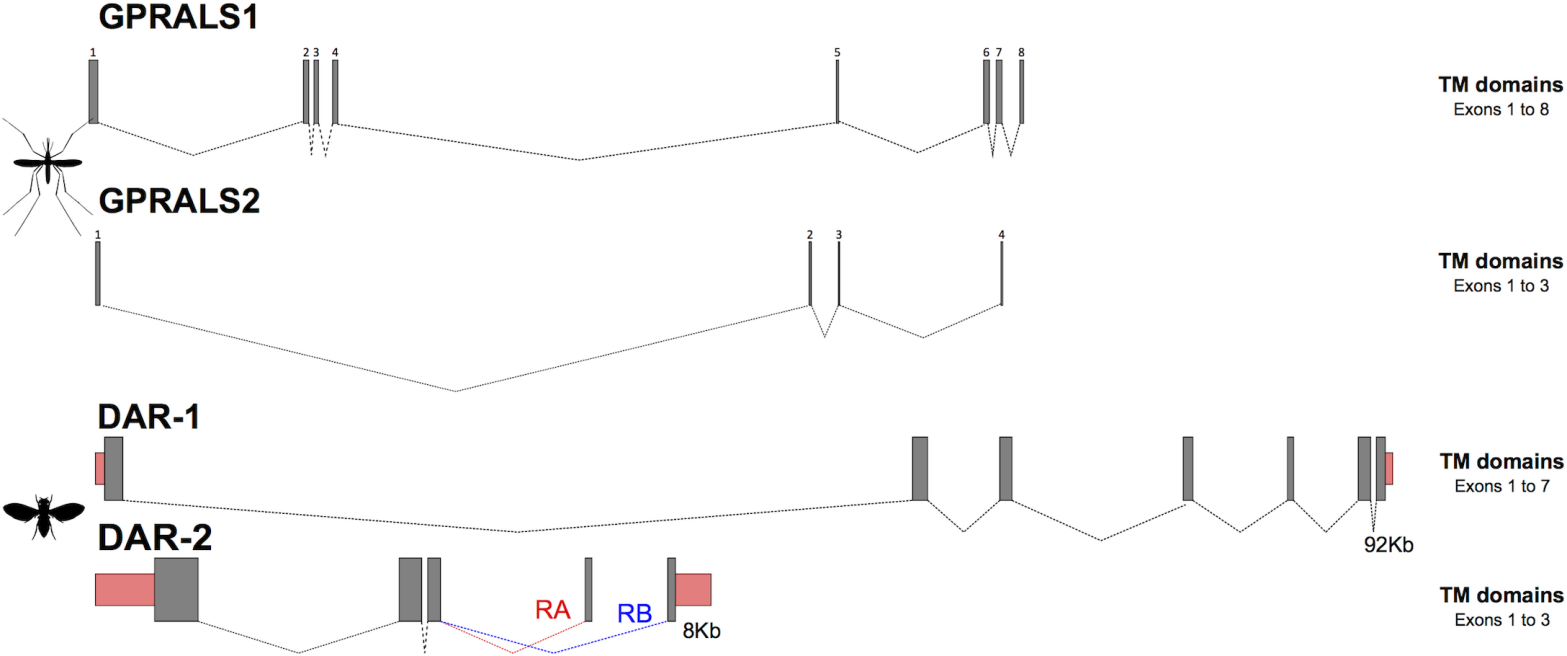
Gene organisation of the AST-A receptors in *Anopheles* and *D*. *melanogaster*. The structure of the *Anopheles* receptor genes was deduced from the consensus organisation obtained from several mosquito genomes (S5 and S6 Tables). The *D*. *melanogaster AST-ARs* gene organizations were obtained from ENSEMBL. In the *A*. *gambiae* PEST genome duplicated exons highly similar in sequence to *GPRALS1* (exon 1) and *GPRALS2* (exon 2, 3 and 4) are predicted and are not represented. Closed boxes represent exons and dashed lines introns. Mosquito exons are numbered and exons encoding the UTR are represented by pink boxes. Gene structures were built using FancyGene 1.4 software. The figure is not drawn to scale.

Characterization of *GPRALS1* in the genomes of other *Anopheles* mosquitoes revealed that receptor gene structure was conserved and that *GPRALS1* was composed of 8 exons and *GPRALS2* of 4 exons (S5 and S6 Tables, Fig 6). The exceptions were the *GPRALS2* genes in two other species of the *A*. *gambiae* complex: *A*. *arabiensis* (Dongola strain) that had duplicated exons 2 and 3 and *A*. *quadriannulatus* (SANGQUA strain) in which exon 2 was duplicated. Furthermore, in common with the *A*. *gambiae* PEST, putative *GPRALS2*-like pseudogenes that map close to *GPRALS2* but had a different orientation (antisense) were identified in *A*. *arabiensis* and *A*. *quadriannulatus* genomes (S6 Table). This seems to be indicative of paracentric inversions, a characteristic of chr 2R evolution [63,65,84].

The amplified *A*. *coluzzii GPRALS1* shared 98% and 99% nucleotide identity, respectively with the predicted *A*. *gambiae* PEST GPRALS1-RA and RB. The nucleotide sequence of the *A*. *coluzzii GPRALS2* was 95% identical to the *A*. *gambiae GPRALS2-RA* and 99% identical to *GPRALS2-RB* and *GPRALS2-RC*. In common with *Anopheles* mosquitoes, the duplicate receptors in *D*. *melanogaster* also had a different gene organisation (Fig 6). The *DAR-1* gene had a higher number of exons than *DAR-2* and the latter receptor gene generated two distinct transcripts via alternative splicing of the last exon [36,38,44].

A single gene that encoded 5 putative AST-A peptides was identified in all *Anopheles* mosquito genomes (data not shown). The AGAP003712 deduced mature AST-A peptides all had a conserved C-terminal FGL-amide motif and were of different lengths: Ano_AST-A1 and Ano_AST-A2 were 8 aa’s; Ano_AST-A3 was 17 aa’s; Ano_AST-A4 was 7 aa’s and Ano_AST-A5 was 9 aa’s (Fig 2) [85]. The *D*. *melanogaster* AST-A precursor (151 aa) generated four peptides (Dme-AST-1 to Dme-AST-4) [16,83,86] and was shorter than the *A*. *gambiae* AST-A precursor (207 aa). The Ano_AST-A1 and Ano_AST-A2 shared 50% aa identity with Dme_AST-A1 and 75% and 87% aa identity with Dme_AST-A2, respectively. Ano_AST-A2 only differed at a single amino acid position (His^3^) from Dme_AST-A2 peptide (Val^3^) (Fig 2). The remaining peptides Ano_AST-A3, Ano_AST-A4 and Ano_AST-A5 shared little similarity with the other *D*. *melanogaster* AST-A peptides.

### Comparisons of the dipteran AST-ARs and AST-A with vertebrate orthologues

**Fig 7:**
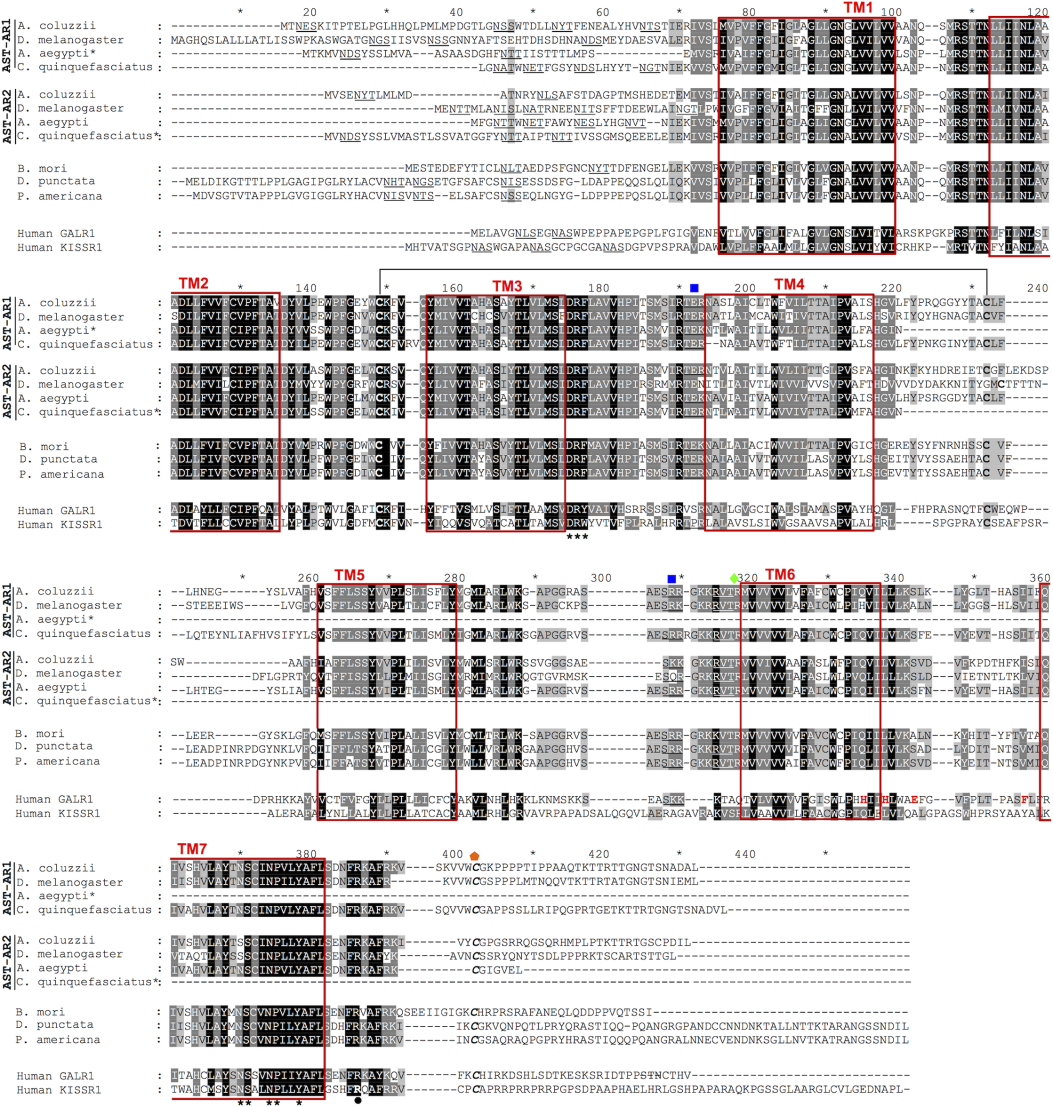
Sequence conservation of the duplicate dipteran AST-ARs with the insect and human orthologues. The predicted seven transmembrane domains are boxed in red and numbered. Potential sites for N-glycosylation are underlined in the N-terminal region and two conserved motifs D-R-Y/F localized after TM3 and NSxxNPxxY within TM7 are annotated with asterisks [87,88]. Two conserved cysteine residues that may form a disulphide bond were identified are connected by a line; predicted residues involved in protein kinase C phosphorylation are indicated by a blue square and potential protein A phosphorylation sites are annotated by a green diamond; C-terminal cysteine residues for potential palmitoylation after TM7 are denoted in italics and indicated with an orange pentagon. Amino acids important for binding of human galanin to GALR1 are indicated in red. The arginine residue important for the function of human KISSR1 that is proximate to the end of TM7 is indicated in bold and circled. Shading denotes amino acid conservation and dark grey means 80% and black 100% conservation. Shading after TM4 was manually edited and did not considered the incomplete receptor regions * indicate incomplete mosquito receptor sequences. Accession numbers of receptor genes are available in S1 Table.

Sequence comparisons of the dipteran AST-ARs were performed with other insects to identify motifs characteristic of the paralogue receptors and similarities with the human orthologues. The duplicate insect AST-ARs shared highly conserved TM domains and divergent N- and C-terminal domains. Both dipteran AST-AR paralogues and other insect AST-ARs shared conserved structural and functional motifs with the vertebrate KISSR1 and GALR1 (Fig 7). This included the seven transmembrane regions and several conserved motifs [87,88]: i) a short N-terminal domain, ii) a DRY/F motif between TM3 and intracellular loop 2, and iii) a NSxxNPxxY motif within TM7. Putative N-glycosylation (N-x-T/S) motifs important for the structure of the N-terminus were also predicted. In addition, the characteristic conserved cysteine residues of the vertebrate KISS/GAL receptors that may form a disulphide bond between ECL1 (between TM2 and TM3) and ECL2 (between TM4 and TM5) were also conserved in insect AST-ARs.

The amino acid motifs important for peptide affinity in GALR, His^264^ in TM6 and His^267^, Glu^271^ and Phe^282^ before TM7, were not conserved in the insect AST-ARs with the exception of His^264^ that was preserved in DAR-1 [89]. However, the Arginine (Arg) residue, localized in the C-terminal region after TM7, that has been linked with the role of the human KISSR1 receptor in precocious puberty, was conserved across all insects [90]. Consensus amino acid signalling motifs responsible for protein kinase C phosphorylation (T/SxR/L), protein kinase A phosphorylation (RxS/T) and the potential palmitoylation cysteine located shortly after TM7, were also conserved between insect AST-ARs and the human orthologues (Fig 7).

**Fig 8:**
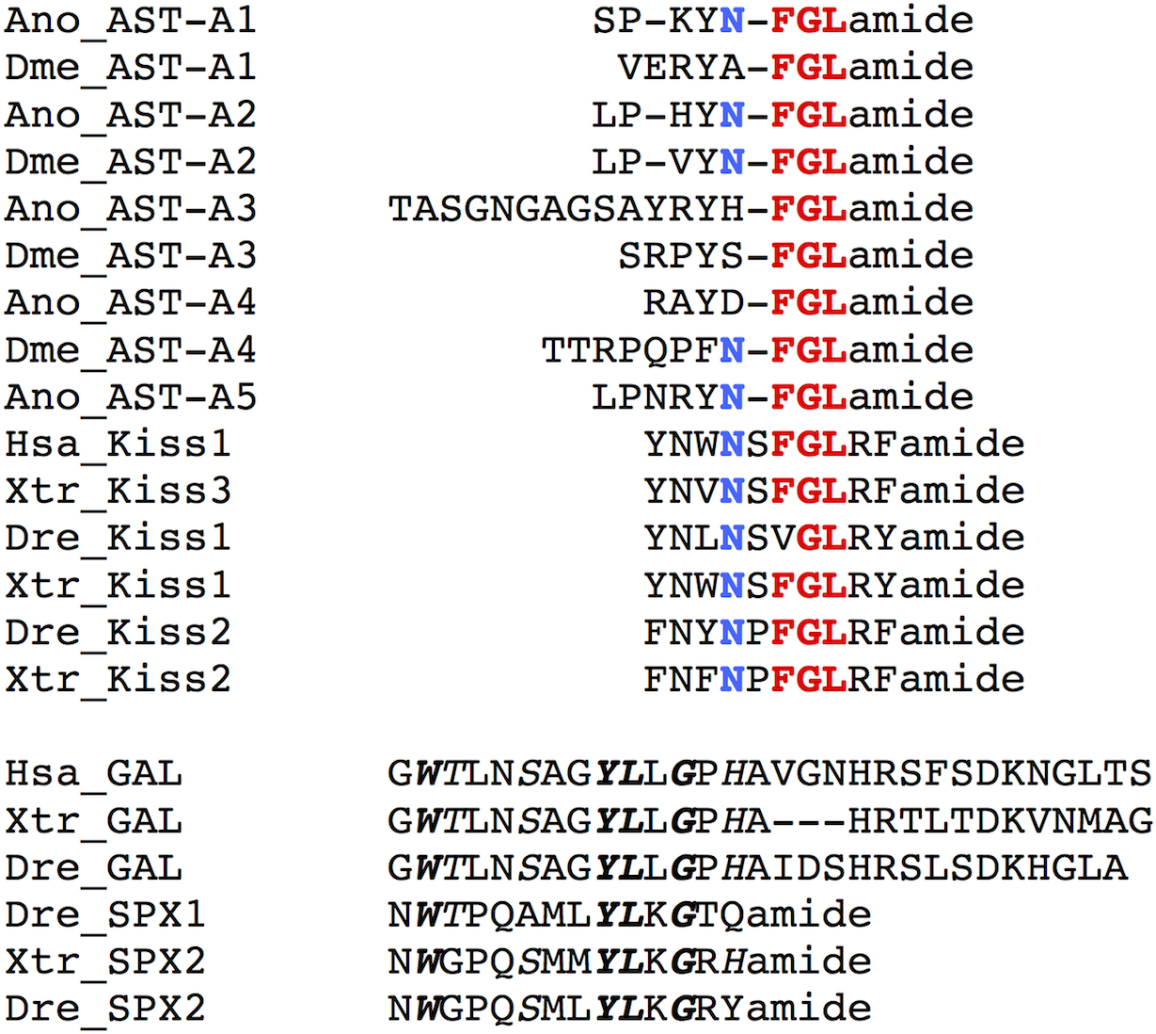
Amino acid sequence alignment of the dipteran AST-A mature peptides with the vertebrate KISS, GAL and SPX family members. The highly conserved FGL motif between AST-A and KISS peptides is indicated in bold and red and conserved N residues in bold and blue. Sequence conservation of GAL and SPX is indicated in italics and totally conserved are in italics and bold. The vertebrate predicted peptide sequences were obtained from [74] and [42] and the *Xenopus laevis* mature galanin peptide deduced from EU446417.1.

The dipteran AST-A peptides shared a C- terminal FGL-amide motif with the vertebrate KISS family and this region is essential for peptide binding and activation of the vertebrate KISSR (Fig 8, [91]). In addition, a conserved Asparagine (Asn) was also found in all insect AST-As and was shared by vertebrate KISS. The vertebrate GAL and SPX peptides shared no sequence conservation with insect AST-A peptides (Fig 8, [42]).

### Tissue expression and effect of blood feeding in the female *A*. *coluzzii*

**Fig 9:**
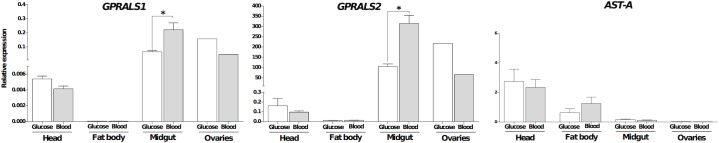
Tissue distribution and effect of a blood meal on the expression of the paralogue *GPRALS* and *AST-A* transcripts in the female *A*. *coluzzii*. Expression was analysed in the head, fat body, midgut and ovaries 3 hours after a glucose (white bars) or blood (grey bars) meals. Receptor expression levels were normalized using the geometric mean of two reference genes (*S7* and *MC*). The results are represented as mean ± SEM of three biological replicates with the exception of ovaries where only a single biological replicate was analysed (~60 ovaries). Prism GraphPad v5 software was used to assess the significance of differences between experimental groups using the Mann-Whitney (two-tailed) test (*p < 0.05).

*GPRALS1* and *GPRALS2* transcripts had an overlapping tissue distribution in *A*. *coluzzii* and were most abundant in the midgut. The relative abundance of *GPRALS2* was approximately >1000 times higher than *GPRALS1* suggesting that receptors may intervene in different functions (Fig 9). Transcripts of both receptors were of very low abundance in the fat body and in the head and of higher abundance in the ovary. Expression of *A*. *coluzzii AST-A* transcripts were also characterized and were most abundant in the head compared to fat body, midgut and ovaries (Fig 9).

After blood feeding, tissue abundance of *AST-ARs* was modified but no difference in abundance of *AST-A* transcripts was detected (Fig 9). *GPRALS1* and *GPRALS2* were both significantly up-regulated in the midgut (*p* < 0.05) and down-regulated in the head and ovaries (statistical analysis in the latter tissue could not be performed as only a single biological replicate of pooled ovaries was used). Expression of carboxypeptidase (*CP*) (*p* < 0.01) and vitellogenin (*Vtg*), well-established markers of physiological events triggered by blood feeding, were up-regulated in whole females 3h after a blood meal compared to glucose fed female mosquitoes (S1 Fig).

### Functional characterisation of the duplicate *A*. *coluzzii* receptors

**Fig 10:**
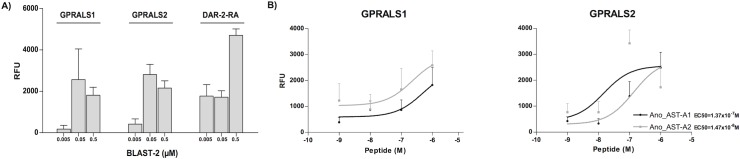
Capacity of the insect AST-A peptides to activate the *A*. *coluzzii* GPRALS. The mosquito Ano_AST-A1 and Ano_AST-A2 peptides and the cockroach BLAST-2 peptide were tested at several different concentrations and the response of the receptor monitored by measuring concentrations of intracellular calcium (RFU). The *D*. *melanogaster* DAR-2-RA receptor was used as a positive control: A) Response to BLAST-2 peptide (0.5 μM to 0.005 μM); B) Receptor response to the presence of decreasing concentrations of Ano_AST-A1 and Ano_AST-A2 peptides (1 μM to 1nM). A Kruskal-Wallis test with a Dunn’s Multiple Comparison test was performed using Prism GraphPad version 5 software. No significant differences were found.

The capacity of AST-A peptides to stimulate GPRALS1 and GPRALS2 was assessed by measuring the cAMP and iCa^2+^ signalling response to *Anopheles* Ano_AST-A1 and Ano_AST-A2 and *B*. *germanica* BLAST-2 peptides. None of the insect peptides induced increased intracellular cAMP but there was a dose dependent increase in iCa^2+^ mobilization (Fig 10). The two *A*. *coluzzii* receptors were activated by *B*. *germanica* BLAST-2 and also by the homologue AST-A peptides, suggesting that they are functional insect AST-A receptors. The potency of Ano_AST-A2 (EC_50_ = 1.47x10^-8^ M) was greater than Ano_AST-A1 (EC_50_ = 1.37x10^-7^ M) for GPRALS2. Both peptides also activated GPRALS1 however, since saturation of the peptide response was not achieved, accurate determination of peptide potency was not possible. Vertebrate GAL (rat 1–29 GAL) and KISS peptides (sea bass KISS 1–10 aa and 2–10 aa) failed to activate *A*. *coluzzii* GPRALS1 and GPRALS2.

## Discussion

AST-As are a functionally important peptide family that regulates development, reproduction and feeding in insects [13,16,25]. In the present study putative AST-ARs were retrieved from the genomes of several arthropods and the origin and evolution of the receptors and their peptide ligands was analysed. The involvement of the duplicate AST-ARs in blood feeding was characterised in *A*. *coluzzii*. Comparative bioinformatics analysis revealed that AST-AR evolution in arthropods was lineage-specific and that the receptors and peptide ligands emerged early in evolution and evolved in parallel with the KISS and GAL family members. Receptor gene duplication occurred in the ancestral bilaterian genome and the invertebrate AST-ARs share the same ancestral gene precursor that originated the KISSR members in lophotrochozoans, early deuterostomes and vertebrate genomes. In dipterans, two AST-AR genes exist and characterization of the AST-AR duplicates revealed that their sequences diverged presumably as a result of different evolutionary pressures. The tissue distribution and abundance of AST-ARs in female *A*. *coluzzii* indicates that they probably acquired different functions but that together they probably integrate feeding and reproduction in common with what occurs in the vertebrate KISS system. We hypothesise that the regulatory function of the ancestral gene has been retained by the AST-A system in protostomes and the KISS system in vertebrates.

### Evolution of AST-AR and AST-A in arthropods was lineage specific

In arthropods, a variable number of *AST-AR* genes and deduced AST-A peptides derived from a unique gene were identified suggesting that both receptors and peptides have evolved by lineage specific events. In the arachnidan *I*. *scapularis*, the branchiopod *D*. *pulex* and the insect *R*. *prolixus* multiple receptors exist and the paralogues are highly related in sequence as the result of species-specific gene duplication. In the other arthropods a single *AST-AR* gene was found. The exceptions were the genomes of *T*. *castaneum* and *D*. *ponderosae* that lack the AST-A system [51,52] and the dipteran genomes where two highly distinct AST-ARs co-exist. The origin of the two Diptera AST-ARs is intriguing and phylogeny and gene structure analysis suggests that after gene duplication the two receptors evolved under distinct evolutionary pressures. The divergence between the dipteran paralogues may be because they arose from a gene duplication event early in the radiation of the insects or that AST-AR gene duplication only occurred in Diptera and suffered considerable modifications in flies and mosquitoes after their divergence (>200 MYA, [46]).

In arthropods, adaptation to different ecological niches has modulated genome evolution and led to differential gene retention [63,84,92–95]. Deletions of *T*. *castaneum AST-AR* and *AST-A* genes may be the result of a species-specific genome rearrangement. The factors underlying the retention of duplicate receptor genes in Diptera and deletion from other arthropod genomes remain to be explained. In *Anopheles* the duplicate AST-ARs map to a fast evolving chromosome (chr 2R) that is under strong natural selection [63,84] and it will be of interest to establish if the same mechanism explains receptor gene number in the genomes of other organisms.

Analysis of several different *Anopheles* genomes suggests that in some species, the *AST-AR* gene structure was modified and that exon tandem duplication and exon inversions occurred during the radiation of *Anopheles*. Modification in *AST-AR* gene structure has mostly affected *GPRALS2* suggesting that speciation modified the evolution of this gene duplicate. Similar mechanisms of gene evolution have also been described for other GPCRs involved in the regulation of development, feeding and reproduction in insects [60]. It was not possible to obtain DNA for the *A*. *gambiae* PEST strain to confirm experimentally the gene structure of AST-ARs and it remains to be established if heterozygosity of the original DNA source led to assembly artefacts that generated additional transcript copies.

The variable number of putative AST-A peptides encoded in each insect gene and their species-specific characteristics has functional implications that remain to be explained [16,24]. For example, in cockroaches the AST-A peptides identified have different potencies for inhibition of JH production by the CA and for stimulation of gut contraction [96,97]. In species with multiple receptor encoding genes, the *AST-A* gene encodes fewer peptides and the inverse is also true. It is tempting to speculate that retention of multiple receptors or peptide encoding genes may be a mechanism to guarantee functional divergence but further studies are required to test this hypothesis.

### AST-A system shared ancestry with KISS and GAL systems

**Fig 11:**
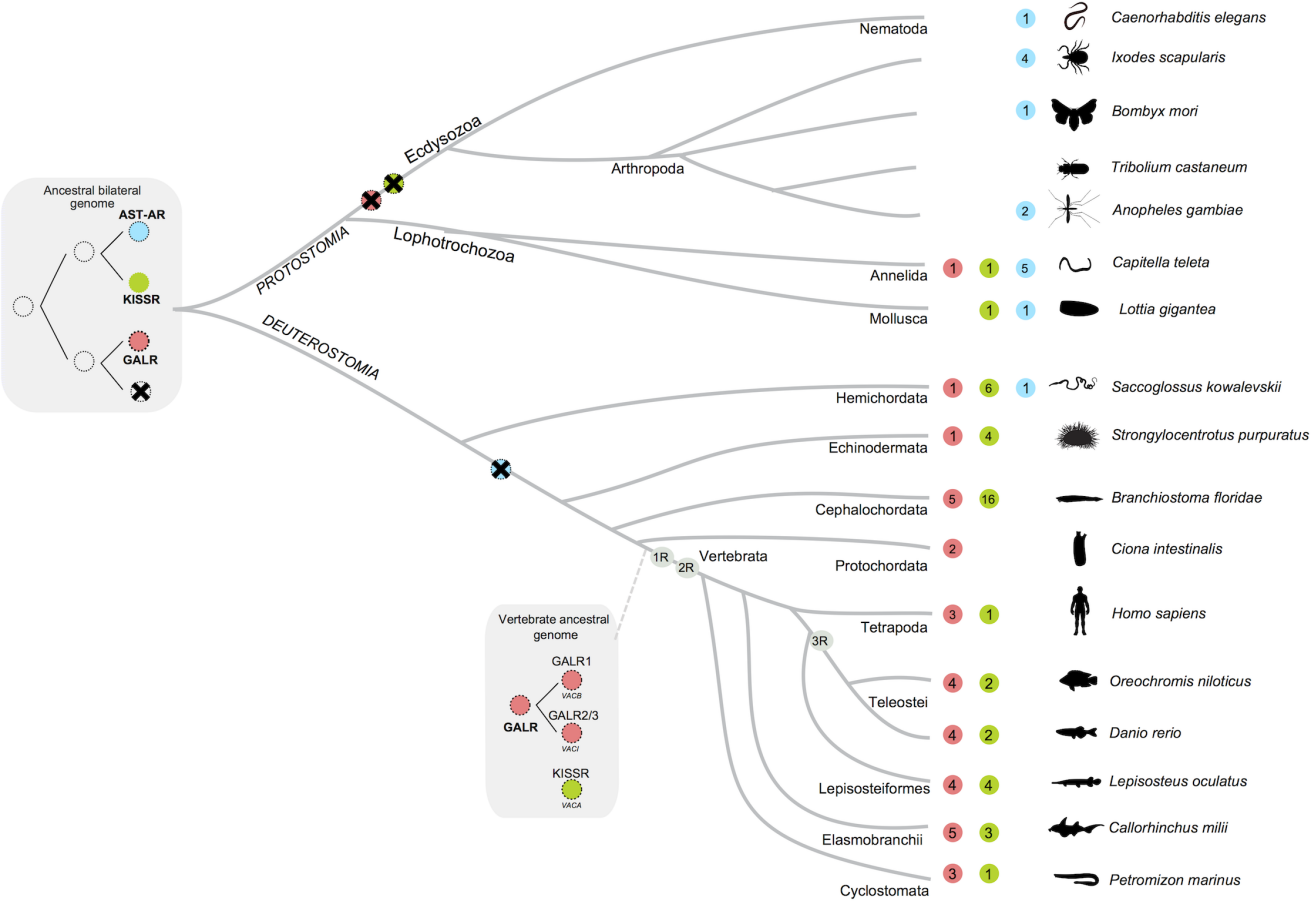
Proposed model for the origin and evolution of the *AST-AR* genes. Circles with different colours represent the AST-AR (light blue), KISSR (green) and GALR (pink) family members. The tetraploidization events basal to vertebrate radiation (1R, 2R) and the teleost specific genome duplication (3R) are indicated. The circle with a cross indicates gene loss during evolution. Numbers within the circles indicate predicted gene numbers of each family. Gene number from early deuterostome and lophotrochozoa representatives were obtained from [41]. For simplicity, lineage-specific duplications are not indicated and the time line is not drawn to scale. Receptor mapping for the vertebrate ancestral chromosomes (VAC) was obtained from [42].

Recently, based upon sequence and gene structure resemblance, eight bilaterian peptidergic signalling systems were identified [41]. Here, using a combination of molecular phylogeny and gene synteny analysis we identified a further peptidergic system and suggest that the invertebrate AST-A and the KISS and GAL family shared a common origin prior to the protostome-deuterostome divergence (Fig 11).

To date the insect AST-ARs and nematode AST-AR-like were considered to be the homologues of the vertebrate GALRs [36,38,39,43]. In fact, sequence similarity searches revealed that AST-AR’s share highest sequence similarity with GALR and the role in feeding and energy metabolism of the AST-A system in nematodes and insects and the GAL system in mammals have been taken as evidence of their functional homology [14,23,36,37,39,98]. The results of the present study suggest that *AST-AR* shared a common evolutionary origin with both *GALRs* and *KISSR* but the phylogenetic analysis and gene synteny analysis suggests that AST-ARs are more related to KISSRs. Identification of *AST-AR*, *KISSR* and *GALR* genes in protostome and deuterostome genomes implies that prior to their divergence the genes emerged. The non-identification of putative *AST-AR* genes in chordates indicates that they were subsequently eliminated from the genome. We propose a new evolutionary model in which the ancestral *AST-AR/KISSR/GALR* gene duplicated and originated the ancestral gene precursor of *AST-AR* and *KISSR* and the ancestral *GALR* gene in the bilaterian genome (Fig 11).

The evolution of the deuterostome KISS and GAL systems has recently been characterised and members of this family were suggested to have emerged prior to the vertebrate radiation. The ancestral *KISSR*, *GALR1* and *GALR2/3* genes were mapped to the vertebrate ancestral chromosome VAC A, VAC B, VAC I, respectively and the current receptor gene repertoire is proposed to have resulted from an early tetraploidization event [42] (Fig 11). However, the existence of putative *KISSR* and *GALR* genes in invertebrate (early deuterostome and lophotrochozoa) genomes [41,99] suggests that the divergence of *KISSR*-like and *GALR*-like genes occurred prior to the divergence of protostomes from deuterostomes. Synteny analysis of the regions flanking *AST-A* genes in protostomes and the *KISS/GAL* paralogon region in human reveals notable conservation and suggests that the peptides also shared a common evolutionary origin. The highly conserved FGL residues in both insect AST-A and vertebrate KISS members and its importance for AST-AR and KISSR activation [46,47,91] also suggests they are closely related.

In vertebrates, KISSR and the respective peptides are key players in reproduction and, in mammals this system acts upstream in the gonadotropic axis mediating gonadotropin secretion and regulation of the onset of puberty [100,101]. KISS peptides are involved in the regulation of the metabolic control of fertility [102,103], regulation of food intake and fat mass production and food restriction increases sex hormone induced KISS1 expression in the adipose tissue of rats [104,105]. The abundance of AST-AR transcripts in the mosquito midgut and ovaries and the changes provoked by a blood meal may indicate that, in addition to molecular conservation with the KISS system, both insect and vertebrate systems may also share conserved physiological roles as integrators of metazoan metabolism and reproduction.

### Functional conservation of the AST-A system across insects

The *A*. *coluzzii* AST-ARs share a similar pharmacological profile with the other insect receptors and activation by cockroach BLAST-2 peptide, that shares the conserved C-terminal FGL-amide, suggests this motif is essential for receptor activation. Despite the limitations of using heterologous expressions systems that inevitably express the receptors out of context our studies revealed that insect AST-A peptides are able to trigger activation of the mosquito receptor and stimulate iCa^2+^ signalling but not cAMP. In contrast in the *corpus cardiacum* of *L*. *migratoria*, *D*. *punctata* AST-2 induced an increase in cAMP [19]. In fact functional characterisation of the AST-AR in several insects revealed that the peptides trigger multiple signalling pathways and that the insect receptors can associate with different members of the G-protein complex [46,47,106]. For full functional characterisation of AST-ARs in *Anopheles* it will be important to also take into consideration the signalling pathways activated by different ligands.

The insect AST-ARs activate similar intracellular signalling pathways to the vertebrate KISSR and GALR homologues but if promiscuous interaction and activation of mosquito GPRALS with human peptides occurs *in vivo* is not yet known. In our study, incubation of GPRALS transfected CHO cells with the vertebrate KISS and GAL peptides (the cognate peptides of the homologue deuterostome receptors) failed to elicit activation of cAMP or iCa^2+^. Nonetheless, before a final conclusion can be reached about GPRALS activation the involvement of other intracellular signalling pathways (eg: PKC-MAPK/ERK) remains to be characterized.

The overall tissue distribution of the duplicate mosquito *GPRALS* and of *AST-A* is similar to other insects and suggests that the AST-A system may play a key role in the regulation of food intake and reproduction [13,14,25]. In *D*. *melanogaster* both peptide and receptors share overlapping tissue distribution and are highly abundant in brain and midgut [11,14,21,86]. In *D*. *punctata*, *AST-AR* is mainly expressed in the brain but it is also expressed in the ovaries and in *B*. *mori* it is mostly detected in the midgut and is of low abundance in the head [11,45,107]. In *R*. *prolixus* AST-A is abundant in the CNS and the receptor is detected in the CNS and in different gut regions [29,46]. The different expression levels of *GPRALS1* and *GPRALS2* in the midgut and ovaries of *A*. *coluzzii* tend to support the notion that they have divergent functions.

In *D*. *melanogaster*, DAR-2 was mainly associated with gut function while DAR-1 was brain specific [36,48,108]. In mosquito the relative importance of the duplicate receptors in physiology remains to be determined. Comparison of the AST-A system between male and female mosquitoes was not carried out in the present study but available data suggests that males express higher levels of the AST-A peptide precursor than females but no differences in receptor expression were found [109]. Future studies will be directed at defining the specific function of the duplicate receptors in male and female mosquitoes.

### Mosquito receptors are responsive to a blood meal

A unique characteristic of the *A*. *gambiae* receptors, in comparison to other insects, is their responsiveness to a blood meal suggesting that participation of the AST-A system in mosquito feeding and reproduction may be triggered by blood feeding. *Anopheles* mosquitoes are anautogenous (feed on blood to reproduce) and during the first hours after a blood meal, the mosquitoes undergo profound physiological, morphological and hormonal changes and significant up-regulation (*p* < 0.05) of AST-ARs occurs. The change in receptor expression levels in tissues involved in blood digestion (midgut, *p* < 0.05) and reproduction (ovaries) suggests that these receptors may be important mediators of the crosstalk between protein digestion and egg maturation in mosquitoes. In *R*. *prolixus* (hematophagous insect) AST-A was proposed to participate in blood digestion as transcript expression was modified by a blood meal and this affected the amount of peptide available for release [29]. In contrast, a blood meal did not modify transcript abundance of *A*. *coluzzii AST-A* transcripts, although it was not possible to assess if peptide levels changed or if feeding modified the ratio of the 5 predicted mosquito AST-A’s. It will be important to carry out further studies that assess peptide levels and the relative importance of the different forms in males and females.

In mosquito females, a blood meal stimulates the secretion of several proteolytic enzymes including carboxypeptidases (CP) from the midgut, which in female mosquitoes is at a higher concentration than in males [110,111]. The nutrient rich (proteins, lipids and carbohydrates) mammalian blood triggers vitellogenesis and restores energy levels for egg development [76,112,113]. Transcription of *Vtg-1*, an egg yolk precursor protein, is triggered by the blood meal and reaches a maximum 24 hours post-feeding [111]. In our study, 3 h post blood feeding, an increase in *CP* occurred relative to glucose fed females (*p* < 0.01) and *Vtg-1* was up-regulated, but to understand the physiological involvement of AST-AR and AST-A in blood protein digestion or egg maturation will require further studies.

## Conclusion

The protostome *AST-AR* and *AST-A* genes emerged prior to the protostome-deuterostome divergence and their similar evolutionary trajectory is suggestive of co-evolution of receptors and their peptides in common with what has been suggested for the metazoan GnRH/AKH, CCK/SK, NMU/PK and Orexin/AT GPCRs and their peptide ligands [40,41,114]. The results of the present study indicate that they evolved in parallel with the KISS and GAL receptor and peptide family members. Evidence is presented that reveals that *AST-AR*, *GALRs* and *KISSR* emerged from a common ancestral gene. Moreover, the analysis performed raises an alternative hypothesis to that which proposes protostome AST-AR as the orthologue of vertebrate GALR. Instead the data indicates that the ancestral gene that originated *AST-AR* also gave rise to *KISSR* and that this occurred after the divergence of a *GALR-like* gene. Duplication of *AST-AR* occurred during the arthropod radiation but in Diptera their divergence in sequence and function indicates they underwent distinct evolutionary pressure. In *A*. *coluzzii*, both receptor transcripts were affected by a blood meal and, it remains to be established if in common with the KISS system in vertebrates they regulate and integrate the link between metabolism and reproduction in insects.

## Supporting Information

S1 Fig Quantitative expression analysis of carboxypeptidase A and vitellogenin1 precursors in glucose fed and blood fed female *A*. *coluzzii*. The results are presented as mean ± SEM of 3 experiments analysed in duplicate. Expression was normalized using the geometric mean of two reference genes (*S7* and *MC*). A Mann-Whitney (two-tailed) test was performed using Prism GraphPad version 5 software to evaluate if differences between groups were significant. Statistical significance is represented with ** (*p* < 0.01).
(PDF)

S1 Table Accession numbers of the predicted AST-AR and AST-A mature proteins and KISSR and GALR used in the study. *scaffolds where genes were deduced; + putative *AST-AR* pseudogenes; # not used for phylogeny. ^**1**^ obtained from [45], ^**2**^ obtained from [67]. The annelid (*C*. *teleta*), mollusc (*L*. *gigantea*), acorn worm (*S*. *kowalevskii*), purple sea urchin (*S*. *purpuratus*), amphioxus (*B*. *floridae*) and tunicate (*C*. *intestinalis*) sequences were obtained from [41].
(PDF)

S2 Table List of primers used for receptor cloning and real-time PCR expression analysis. (PDF)

S3 Table Human (*H*. *sapiens*), *D*. *melanogaster*, *T*. *castaneum* and *C*. *elegans* genes orthologues of the *A*. *gambiae GPRALS1* and *GPRALS2* gene environment on chr 2R. Accession numbers, chromosome positions, symbol and initial gene positions (base pair) are given. The data was extracted using Ensembl Biomart software and confirmed using sequence similarity searches.
(PDF)

S4 Table Human (*H*. *sapiens*), *D*. *melanogaster*, *T*. *castaneum* and *C*. *elegans* gene orthologues of the *A*. *gambiae AST-A* gene environment on chr 2R. Accession numbers, chromosome position, symbols and initial gene positions (base pair) are given. The data was extracted using Ensembl Biomart software and confirmed using sequence similarity searches.
(PDF)

S5 Table Gene organisation of the *GPRALS1* in *Anopheles* mosquitoes. Mosquito genomes were accessed in VectorBase (https://www.vectorbase.org/, March 2015) and receptor gene structure was deduced by homology with the *GPRALS1* transcript. The number and approximate size of the deduced exons (E) and introns (I) are given in base pairs (bp). E5 of *A*. *gambiae* PEST is not predicted in the reference genome and was deduced by homology. E1 is duplicated in *A*. *gambiae* PEST. *Anopheles* species in which sequence hits were found to short genome scaffolds are not represented. ni—not identified, * incomplete sequences.
(PDF)

S6 Table Gene organisation of the *GPRALS2* in *Anopheles* mosquitoes. Mosquito genomes were accessed in VectorBase (https://www.vectorbase.org/, March 2015) and receptors gene structures were deduced by homology with the *GPRALS2* transcript. The predicted transcripts based on genome annotation are also indicated. The number and approximate size of the exons (E) and introns (I) are given in base pairs (bp). Duplicate exons and inverted exons were found for *Anopheles* species (*A*. *gambiae* PEST strain, *A*. *arabiensis* Dongola strain and *A*. *quadriannulatus* SANGQUA strain) suggesting that alternative receptor transcripts may exist. *Anopheles* species in which sequence hits were found to short scaffolds are not represented. The predicted gene structure of *A*. *coluzzii* (Yaoundé strain) gene is presented and was deduced by homology with the *A*. *coluzzii* (MALI-NIH strain) and I2 size was estimated based on PCR of genomic DNA (data not shown). ni—not identified. * incomplete sequences. Transcripts that cover similar exon regions are in italics.
(PDF)
