# Supplementary figures and images for: Unravelling the Evolution of the Allatostatin-Type A, KISS and Galanin Peptide-Receptor Gene Families in Bilaterians: Insights from Anopheles Mosquitoes
Source: PLoS One. 2015 Jul 2;10(7):e0130347. doi: 10.1371/journal.pone.0130347 (PMC4489612; doi:10.1371/journal.pone.0130347)

**CP**

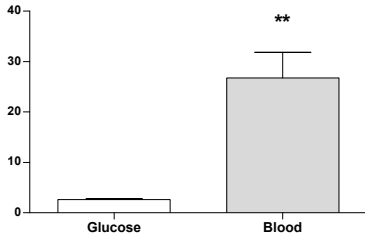

**Vgt**

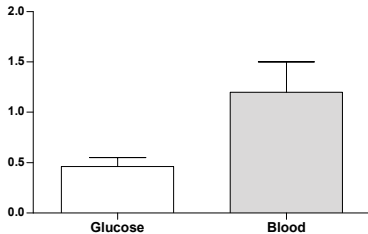

Supplement: S1 Fig — The results are presented as mean ± SEM of 3 experiments analysed in duplicate. Expression was normalized using the geometric mean of two reference genes (S7 and MC). A Mann-Whitney (two-tailed) test was performed using Prism GraphPad version 5 software to evaluate if differences between groups were significant. Statistical significance is represented with ** (p < 0.01). (PDF) [file pone.0130347.s001.pdf]
