# Supplementary material for: Unravelling the Evolution of the Allatostatin-Type A, KISS and Galanin Peptide-Receptor Gene Families in Bilaterians: Insights from Anopheles Mosquitoes
Source: PLoS One. 2015 Jul 2;10(7):e0130347. doi: 10.1371/journal.pone.0130347 (PMC4489612; doi:10.1371/journal.pone.0130347)
Supplement: S1 Table — *scaffolds where genes were deduced; + putative AST-AR pseudogenes; # not used for phylogeny. 1 obtained from [45], 2 obtained from [67]. The annelid (C. teleta), mollusc (L. gigantea), acorn worm (S. kowalevskii), purple sea urchin (S. purpuratus), amphioxus (B. floridae) and tunicate (C. intestinalis) sequences were obtained from [41]. (PDF) [file pone.0130347.s002.pdf]

| | AST-AR | AST-A | GALR | KISSR |
|---|---|---|---|---|
| ***PROTOSTOMES*** | | | | |
| *ARTHROPODA* | | | | |
| *I. scapularis* | ISCW001334<br>ISCW0016381<br>ISCW016382<br>ISCW014938 | ISCW022939 | | |
| *T. urticae* | tetur17G01230 | tetur06g00870 | | |
| *D. pulex* | EFX62248<br>EFX71121<br>EFX75149 | EFX87432 | | |
| *D. punctata* | Ast-R[1] | P12764.2 | | |
| *P. americana* | AAK52473.1 | CAA62500.1 | | |
| *P. humanus* | PHUM343230 | PHUM231810 | | |
| *R. prolixus* | RPRC004706<br>RPRC004708<br>RPRC004705+# | ACX47066 | | |
| *A. pisum* | ACYPI008623 | ACYPI24660 | | |
| *A. cephalotes* | ACEP10785 | DAA35078 (7) | | |
| *S. invicta* | SINV18345 | Scaffold03503* | | |
| *N. vitripennis* | NV10415 | NV21997 | | |
| *A. mellifera* | GB43574 | GB47928 | | |
| *B. mori* | BGIBMGA005708 | BGIBMGA014377 | | |
| *D. plexippus* | EHJ71577 | EHJ64275 | | |
| *H. melpomene* | HMEL006667 | HMEL014728 | | |
| *A. aegypti* | AAEL007169<br>AAEL006076<br>AAEL006077+# | AAEL015251 | | |
| *C. quinquefasciatus* | Supcontig3.374*<br>CPIJ013095<br>CPIJ011118+# | CPIJ008017 | | |
| *A. darlingi* | Scaffold_325*#<br>Scaffold_130*<br>Scaffold_4146+# | ADAC004794 | | |
| *A. gambiae* | AGAP003658<br>AGAP001773<br>AGAP001774+# | AGAP003712 | | |
| *D. melanogaster* | FBgn0028961<br>FBgn0039595 | FBgn0015591 | | |
| *M. scalaris* | C443560*<br>MESCA004796+# | *ni* | | |
| *NEMATODA* | | | | |
| *C. elegans* | ZK455.3 | | | |
| *ANNELIDA* | | | | |
| *C. teleta* | ELT93650<br>ELU05381<br>ELU16355<br>ELT93995 | | ELU13887 | ELU15450 |
| *MOLLUSCA* | | | | |
| *L. gigantea* | ESO84146 | | | ESO82200 |
| ***DEUTEROSTOMES*** | | | | |
| *HEMICHORDATA* | | | | |
| *S. kowalevskii* | XP_002730936 | | XP_002731810 | XP_00681189<br>NP_001161574<br>XP_002735795 |
| *ECHINODERMATA* | | | | |
| *S. purpuratus* | | | SPU_022317 | XP_00787561<br>XP_00784787<br>XP_00796286 |
| *CEPHALOCHORDATA* | | | | |
| *B. floridae* | | | XP_002609164<br>XP_002604982<br>EEN60991 | EEN47971<br>XP_002611682<br>XP_002613701 |
| *TUNICATA* | | | | |
| *C. intestinalis* | | | XP_002122839<br>XP_002120122 | |
| *VERTEBRATA* | | | | |
| *P. marinus* | | | ENSPMAP00000009466<br>ENSPMAP00000000353<br>ENSPMAP00000002955 | ENSPMAP00000001601 |
| *C. milli* | | | SINCAMP00000007123<br>XP_007898764<br>XP_007886989<br>XP_0079003060 | XP_007898035<br>XP_007906481<br>XP_007906256 |

| | | |
|---|---|---|
| *O. niloticus* | XP_007907774<br>XP_003447310<br>XP_003443686<br>XP_003442232 | NP_001266708<br>XP_005459884 |
| *D. rerio* | XP_00344874<br>XP_696215.1<br>ENSDARP00000111929<br>XP_002664053 | XP_005163556<br>ADV33377 |
| *L. oculatus* | XP_001339169<br>XP_00664158,<br>XP_006635729<br>XP_006637044 | ENSLOCP00000002832<br>ENSLOCP00000003036<br>ENSLOCP00000002856 |
| *L. chalumnae* | XP_006635498<br>XP_006001469<br>XP_006012914<br>XP_005993296 | ENSLOCP00000008391<br>KissR-1[2]<br>KissR-2[2]<br>KissR-3[2] |
| *X. tropicalis* | XP_006013200<br>NP_00107355<br>NP_001096443<br>XP_002934279 | KissR-4[2]<br>NP_001165296<br>NP_001163985<br>NP_001165295 |
| *A. carolinensis* | XP_002940025<br>XP_003219765<br>XP_003217293 | XP_00321788 |
| *G. gallus* | XP_03221019<br>NP_001121534<br>NP_001122137<br>NP_001121535 | |
| *H. sapiens* | NP_001124057<br>NP_001471<br>NP_003848<br>NP_003605 | AAK83235 |
