# Supplementary material for: Unravelling the Evolution of the Allatostatin-Type A, KISS and Galanin Peptide-Receptor Gene Families in Bilaterians: Insights from Anopheles Mosquitoes
Source: PLoS One. 2015 Jul 2;10(7):e0130347. doi: 10.1371/journal.pone.0130347 (PMC4489612; doi:10.1371/journal.pone.0130347)
Supplement: S2 Table — (PDF) [file pone.0130347.s003.pdf]

	5'-3'	T°C	Efficiency (r^2)
**Receptor isolation**			
*GPRALS1*fwd	atgacgaacgagtctaaaatta	60	
*GPRALS1*rev	tcataaggggtccgcgttcga		
*GPRALS2*fwd	atggtaagcgagaactacacgc	60	
*GPRALS2*rev	ttataaaatgtctggacagctg		
**q-PCR**			
*S7*fwd	cattctgcccaaaccgatgcgt	64	102% (r^2=0.99)
*S7*rev	cgggaataccagatcctccagg		
*MC*fwd	cctgcccacgacggtcatttactt		
*MC*rev	cgtcccgccctttccttctttg		103% (r^2=0.99)
*Vtg*fwd	acgaaaaccatgaccgctct	60	103% (r^2=0.99)
*Vtg*rev	cttgggacggatcaccaaat		
*CP*fwd	cgttctcggaggttgagac	60	99.5% (r^2=0.99)
*CP*rev	ctgcgagtacgagtggaacg		
*GPRALS1*fwd	ggttgccgcgaaccagag	60	106% (r^2=0.99)
*GPRALS1*rev	ccaatactctccgaagggc		
*GPRALS2*fwd	gtccaacccacagatgcg	56	84% (r^2=0.95)
*GPRALS2*rev	gacgctcttgcaccatagc		
*AST-A*fwd	gctacatcattgaggacgtgcg	58	104%(r^2=0.99)
*AST-A*rev	atcagtccatcgtactcgtaat		
