# Supplementary material for: Unravelling the Evolution of the Allatostatin-Type A, KISS and Galanin Peptide-Receptor Gene Families in Bilaterians: Insights from Anopheles Mosquitoes
Source: PLoS One. 2015 Jul 2;10(7):e0130347. doi: 10.1371/journal.pone.0130347 (PMC4489612; doi:10.1371/journal.pone.0130347)
Supplement: S3 Table — Accession numbers, chromosome positions, symbol and initial gene positions (base pair) are given. The data was extracted using Ensembl Biomart software and confirmed using sequence similarity searches. (PDF) [file pone.0130347.s004.pdf]

Human				*A. gambiae*				*D.melanogaster*				*C. elegans*				*T. castaneum*			
Gene	chr	symbol	initial position (pb)	Gene	chr	symbol	initial position (pb)	Gene	chr	symbol	initial position(bp)	Gene	chr	symbol	initial position (pb)	Gene	chr	symbol	initial position (pb)
Polypyrimidine tract binding protein (PTBP)																			
ENSG00000011304	19	PTBP1	797075	AGAP003945	2R		46708274	FBgn0011224	3R		31846987					TC011728	9		19360223
ENSG00000117569	1	PTBP2	96721665																
ENSG00000119314	9	PTBP3	112217716																
Allatostatin/KISSR																			
**ENSG00000116014**	**19**	**KISS1R**	**917287**	**AGAP003658**	**2R**	**GPRALS1**	**41239013**	**FBgn0028961**	**X**	**DAR-1**	**3574536**	**WBGene00013974**	**X**	**NPR-9**	**11328591**				
				**AGAP001773**	**2R**	**GPRALS2**	**9766909**	**FBgn0039595**	**3R**	**DAR-2**	**28737109**								
				AGAP001774	2R		10041418												
ecotropic viral integration site 5 proteins (EVI5)																			
ENSG00000142459	19	EVI5L	7830233	AGAP002354	2R		20571876	FBgn0262740	X		11430523	WBGene00020770	X		16399210	TC002602	3		24800274
ENSG00000067208	1	EVI5	92508696																
DOT1-like histone H3K79 methyltransferase proteins (DOT1L)																			
ENSG00000104885	19	DOT1L	2164149	AGAP003282	2R		34856256	FBgn0264495	3R		6406855	WBGene00022512	X		1917469	TC013593	X		4314300
												WBGene00010067	X		11867971				
												WBGene00010120	X		11929899				
												WBGene00012302	X		12301590				
outer dense fiber of sperm tails 3 protein (ODF3L)																			
ENSG00000104885	19	ODF3L2	43346	AGAP001620	2R		6966595	FBgn0039104	3R		23695221					TC009948	7		14413903
				Homeobox protein unc-4															
				AGAP001495	2R	unc-4	5610198	FBgn0024184	X		17768569								
				wide awake (wake)															
				AGAP001726	2R		8941088	FBgn0266418	3R	wake	22639808	WBGene00019935	X		5959115	TC005383	8		17302996
				string (stg)															
				AGAP012963	2R		36867688	FBgn0064123	X	stg	21093844					TC006018	8		2899353
