# Supplementary material for: Unravelling the Evolution of the Allatostatin-Type A, KISS and Galanin Peptide-Receptor Gene Families in Bilaterians: Insights from Anopheles Mosquitoes
Source: PLoS One. 2015 Jul 2;10(7):e0130347. doi: 10.1371/journal.pone.0130347 (PMC4489612; doi:10.1371/journal.pone.0130347)
Supplement: S4 Table — Accession numbers, chromosome position, symbols and initial gene positions (base pair) are given. The data was extracted using Ensembl Biomart software and confirmed using sequence similarity searches. (PDF) [file pone.0130347.s005.pdf]

Human				***A. gambiae***				***D. melanogaster***				***C. elegans***				***T. castaneum***			
Gene	chr	symbol	initial position (bp)	Gene	chr	symbol	initial position (bp)	Gene	chr	symbol	initial position (bp)	Gene	chr	symbol	initial position (bp)	Gene	chr	symbol	initial position (bp)
inward rectifying potassium channel superfamily (KCNJ)																			
ENSG00000162728	1	KCNJ9	160081570	AGAP001280	2R		2263694	FBgn0039061	3R		23168317	WBGene0002149	X		6766579	TC006706	8		16260797
ENSG00000177807	1	KCNJ10	160037467	AGAP001281	2R		2268140	FBgn0039081	3R		23513617	WBGene00002150	X		702974	TC006707	8		16270824
ENSG00000121361	12	KCNJ8	21764955	AGAP001283	2R		2276105												
ENSG00000182324	19	KCNJ14	48455509	AGAP001284	2R		2280076												
ENSG00000187486	11	KCNJ11	17385859																
ENSG00000151704	11	KCNJ1	128836315																
ENSG00000120457	11	KCNJ5	128891356																
LAR protein-tyrosine phosphatase-interacting protein (PPFIA)																			
ENSG00000143847	1	PPFIA4	203026498	AGAP001469	2R		5341315					WBGene00006364	X		10549123	TC012918	2		3628762
ENSG00000139220	12	PPFIA2	81257975									WBGene00011904	X		13865038				
ENSG00000177380	19	PPFIA3	49119389					FBgn0034720	2R		22333500								
ENSG00000131626	11	PPFIA1	70270700																
Golgi Transport (GOLT)																			
ENSG00000174567	1	GOLT1A	204198160	AGAP002357	2R		20595150					WBGene00018270	II		4740410				
ENSG00000111711	12	GOLT1B	21501781																
glycogen synthase (GYS)																			
ENSG00000111713	12	GYS2	21536189																
ENSG00000104812	19	GYS1	48968125	AGAP002586	2R		23292023	FBgn0038293	3R		15141843	WBGene00001793	II		12866448	TC015651	6		6648641
Allatostatin/Kiss/Galanin/Spexin (AST,KISS,GAL,SPX)																			
**ENSG00000170498**	**1**	**KISS1**	**204190341**	**AGAP003712**	**2R**	**ASTA**	**42302848**	**FBgn0015591**	**3R**	**ASTA**	**24760530**	**WBGene00003744**	**X**	**NLP-6**	**17662458**				
ENSG00000069482	11	GAL	68683779																
ENSG00000134548	12	SPX	21526307																
aspartic protease family (REN, NAPSA, CTSD, CathD)																			
ENSG00000143839	1	REN	204154819	AGAP003277	2R		34793432	FBgn0029093	2R		7822111	WBGene00000216	X		6791064	TC004989	2		3124353
ENSG00000143839	19	NAPSA	50358477					FBgn0033933	2R		14390696	WBGene00000217	X		13218977				
ENSG00000117984	11	CTSD	1752752																
N-acylneuraminate cytidylyltransferase (CMAS)																			
ENSG00000111726	12	CMAS	22046174	AGAP004527	2R		57391338									TC015995	8		1531685
				AGAP012963	2R		36867688	FBgn0064123	X	stg-1	21093844					TC006018	8		2899353
