# Supplementary material for: Unravelling the Evolution of the Allatostatin-Type A, KISS and Galanin Peptide-Receptor Gene Families in Bilaterians: Insights from Anopheles Mosquitoes
Source: PLoS One. 2015 Jul 2;10(7):e0130347. doi: 10.1371/journal.pone.0130347 (PMC4489612; doi:10.1371/journal.pone.0130347)
Supplement: S5 Table — Mosquito genomes were accessed in VectorBase (https://www.vectorbase.org/, March 2015) and receptor gene structure was deduced by homology with the GPRALS1 transcript. The number and approximate size of the deduced exons (E) and introns (I) are given in base pairs (bp). E5 of A. gambiae PEST is not predicted in the reference genome and was deduced by homology. E1 is duplicated in A. gambiae PEST. Anopheles species in which sequence hits were found to short genome scaffolds are not represented. ni—not identified, * incomplete sequences. (PDF) [file pone.0130347.s006.pdf]

Species	Strain	Chromosome/ Scaffold	Predicted Transcripts	E1	I1	E2	I2	E3	I3	E4	I4	E5	I5	E6	I6	E7	I7	E8
Anopheles gambiae	PEST	2R	AGAP003658-RA<br>AGAP003658-RB	**247**<br>**247**	784<br>18189	**155**	141	**131**	393	**148**	16596	**120**	6941	**63**	71	**165**	502	**105**
	Pimperena/S-form	scf_1106392397132		**247**	7284	**158**	139	**131**	399	**150**	17612	**126**	7259	**82**	67	**161**	495	**107**
Anopheles coluzzii	Mali-NIH	scf_1925491385	ACOM028757-RA*	**247**	7225	**158**	139	**131**	402	**150**	17176	**126**	6867	**82**	67	**161**	584	**107**
Anopheles albimanus	STECLA strain	KB672446	AALB009020-RA*<br>AALB009019-RA*	**ni**	ni	**158**	85	**128**	116	**150**	13124	**123**	3567	**81**	94	**161**	299	**107**
Anopheles arabiensis	Dongola	KB704407	AARA010137-RA*<br>AARA010136-RA*<br>AARA010135-RA*	**247**	5407	**158**	136	**131**	396	**150**	17575	**126**	6576	**82**	67	**161**	506	**107**
Anopheles atroparvus	EBRO	scf_1925491385	AATE010991-RA*<br>AATE011451-RA*	**133***	5123	**158**	135	**128**	363	**150**	15709	**123**	ni	**ni**	ni	**161**	540	**110**
Anopheles culicifacies A	A-37	KI422474	ACUA006292-RA*<br>ACUA003786-RA*	**237**	5072	**158**	106	**128**	319	**149**	16734	**120**	3723	**81**	79	**160**	377	**107**
Anopheles dirus A	WRAIR2	KB672713	ADIR005565-RA*<br>ADIR005564-RA*	**243**	6451	**158**	76	**128**	399	**149**	27818	**120**	7019	**86**	70	**162**	431	**107**
Anopheles epiroticus	Epiroticus2	KB670724	AEPI001641-RA*<br>AEPI001640-RA*	**247**	5138	**158**	118	**128**	340	**150**	12959	**120**	4515	**81**	88	**162**	490	**107**
Anopheles farauti	FAR1	KI421658	AFAF007839-RA*<br>AFAF005284-RA*	**242**	7104	**158**	87	**128**	396	**140**	21328	**120**	6834	**82**	77	**161**	533	**107**
Anopheles funestus	FUMOZ	KB668761	AFUN003461-RA*<br>AFUN003462-RA*	**237**	4686	**158**	100	**128**	369	**150**	14906	**123**	ni	**ni**	ni	**160**	419	**107**
Anopheles merus	MAF	AXCQ01003868	AMEM003822-RA*<br>AMEM010034-RA*	**ni**	ni	**158**	144	**131**	399	**150**	16673	**126**	6026	**82**	67	**161**	504	**107**
Anopheles minimus A	MINIMUS	KB663610	AMIN004016-RA*<br>AMIN004017-RA*	**238**	5206	**158**	91	**128**	354	**149**	16342	**120**	4309	**82**	76	**161**	396	**107**
Anopheles quadriannulatus	SANGQUA	KB665398	AQUA001555-RA*<br>AQUA001554-RA*	**247**	6096	**158**	135	**131**	405	**150**	16266	**126**	7133	**82**	67	**161**	501	**107**
Anopheles sinensis	China	AS2_scf7180000695652	ASIC005793-RA*<br>ASIC005790-RA*	**196***	4716	**158**	96	**128**	324	**143**	12407	**123**	5553	**81**	183	**161**	415	**107**
Anopheles stephensi	Indian	scaffold_00054	ASTEI07423-RA*<br>ASTEI07424-RA*	**247**	6576	**158**	94	**128**	355	**149**	15659	**128**	3753	**84**	80	**161**	415	**113**
	SDA-500	KB664622	ASTE001379-RA*<br>ASTE001380-RA*	**247**	6561	**158**	94	**128**	355	**149**	15951	**128**	3751	**84**	80	**161**	415	**113**
