# Supplementary material for: Unravelling the Evolution of the Allatostatin-Type A, KISS and Galanin Peptide-Receptor Gene Families in Bilaterians: Insights from Anopheles Mosquitoes
Source: PLoS One. 2015 Jul 2;10(7):e0130347. doi: 10.1371/journal.pone.0130347 (PMC4489612; doi:10.1371/journal.pone.0130347)
Supplement: S6 Table — Mosquito genomes were accessed in VectorBase (https://www.vectorbase.org/, March 2015) and receptors gene structures were deduced by homology with the GPRALS2 transcript. The predicted transcripts based on genome annotation are also indicated. The number and approximate size of the exons (E) and introns (I) are given in base pairs (bp). Duplicate exons and inverted exons were found for Anopheles species (A. gambiae PEST strain, A. arabiensis Dongola strain and A. quadriannulatus SANGQUA strain) suggesting that alternative receptor transcripts may exist. Anopheles species in which sequence hits were found to short scaffolds are not represented. The predicted gene structure of A. coluzzii (Yaoundé strain) gene is presented and was deduced by homology with the A. coluzzii (MALI-NIH strain) and I2 size was estimated based on PCR of genomic DNA (data not shown). ni—not identified. * incomplete sequences. Transcripts that cover similar exon regions are in italics. (PDF) [file pone.0130347.s007.pdf]

Species	Strain	Chromosome/ Scaffold	Predicted Transcripts	E1	I1	E2	I2	E3	I3	E4
Anopheles gambiae	PEST	2R	AGAP001773-RA	**549**	201254	**240**	2834	**159**	10241	**102**
		(sense)	AGAP001773-RB	**549**	128245	**270**	3373	**159**	10416	**102**
			AGAP001773-RC	**549**	42200	**270**	5121	**159**	10099	**102**
		2R (antisense)	AGAP001774-RA	**ni**	ni	**270**	3443	**59**	10045	**102**
Anopheles coluzzii	Yaoundé		Our study	**549**	ni	**272**	~3500	**157**	ni	**102**
Anopheles arabiensis	Dongola	KB704673	AARA002223-RA*	**548**	49291	**239**	3304	**171**	10008	**104**
		(sense)	*AARA002224-RA**		130556	**228**	3450	**171**		
			*AARA002225-RA**							
		KB704673	*AARA002221-RA**	ni	ni	**272**	3327	**171**	ni	**ni**
		(antisense)	*AARA002222-RA**			**277**	4051	**171**		
Anopheles atroparvus	EBRO	KI421888	AATE013630-RA*	**537**	30418	**213**	4752	**162**	17124	**104**
			AATE019696-RA*							
Anopheles darling	Coari	Scaffold_130	ni	**542**	33527	**240**	3601	**163**	10335	**104**
Anopheles dirus A	WRAIR2	KB672868	ADIR003427-RA*	**549**	46683	**272**	3989	**162**	12706	**104**
			ADIR003426-RA*							
Anopheles minimus A	MINIMUS	KB663610	AMIN003195-RA*	**549**	40039	**272**	3383	**162**	11489	**104**
			AMIN003194-RA*							
Anopheles quadriannulatus	SANGQUA	KB667500	AQUA004119-RA*	**548**	56495	**271**	3356	**175**	10281	**104**
		(sense)	*AQUA004120-RA**		198383	**264**				
			*AQUA004121-RA**							
		KB667500	AQUA004118-RA*	**119***	ni	**272**	ni	**ni**	ni	**ni**
		(antisense)		**119***	ni	**272**				
Anopheles sinensis	China	KI398091	ASIS005833-RA*	**537**	24787	**267**	1616	**163**	8399	**104**
			ASIS011825-RA*							
Anopheles stephensi	Indian	Scaffold_00002	ASTEI00480-RA*	**545**	39899	**270**	3687	**162**	17119	**104**
			ASTEI00481-RA*							
	SDA-500	KB664432	ASTE010678-RA*	**545**	38835	**270**	3686	**162**	12642	**104**
			ASTE010679-RA*							
